# Development of long short-term memory models using rainfall and soil moisture to predict soil moisture dynamics

**DOI:** 10.1371/journal.pone.0353268

**Published:** 2026-07-21

**Authors:** Yoon Ji Kim, Ho Jin Im, Seung Gon Wi, Min Soo Kim, Seok Min Lee, Ung Yang, Sun Woo Chung, Sang-Hyun Lee

**Affiliations:** 1 Department of Horticulture, Chonnam National University, Gwangju, Republic of Korea; 2 Interdisciplinary Program in IT-Bio Convergence System, Chonnam National University, Gwangju, Republic of Korea; 3 Department of Statistics, Chonnam National University, Gwangju, Republic of Korea; 4 Digital Agriculture Research Institute for Horticultural Fruit, Chonnam National University, Gwangju, Republic of Korea; 5 Gyeongsangnam-do Agricultural Research and Extension Services, Jinju, Republic of Korea; 6 Pear Research Center, National Institute of Horticultural & Herbal Science, Rural Development Administration, Naju, Republic of Korea; Swedish Meteorological and Hydrological Institute, SWEDEN

## Abstract

Long short-term memory (LSTM) models were developed using rainfall and soil moisture data to predict soil moisture at various depths in an Asian pear orchard. Two types of rainfall inputs were tested: hourly rainfall data treated as individual values and event-based rainfall data, where cumulative rainfall was calculated by applying the minimum inter-event time threshold (12 h). Soil moisture was measured at depths of 20, 40, and 60 cm from the soil surface during 2023 and 2024 using frequency domain reflectometry. The models were trained to predict soil moisture at time horizons of ‘t + 1’, ‘t + 3, ‘t + 6’, and ‘t + 12’ when ‘t’ is present time. Short-term predictions more accurately followed the observed soil moisture trends than long-term predictions. During the collection period, rainfall varied from 0.5 mm to 48.5 mm per hour. Soil moisture contents increased immediately following the onset of rainfall. These results are consistent with existing knowledge. As soil depth increased, soil moisture contents tended to increase and respond more gradually to rainfall. During rainfall, in the topsoil (20 cm depth) moisture content fluctuated significantly, while the subsoil (60 cm depth) remained relatively stable for several hours after rainfall ended. The model performance was evaluated using mean absolute error, root mean square error (RMSE), and normalized RMSE values. In both models using hourly and event-based rainfall inputs, errors in soil moisture prediction tended to increase with longer forecast time horizons. However, these errors were significantly lower when using event-based rainfall data. These findings indicate that event-based rainfall is a more effective input for LSTM models in predicting soil moisture in an Asian pear orchard, and such models can support precision irrigation systems that optimize water use by delivering the right amount of water at the right time.

## Introduction

Soil moisture, the amount of water contained in unsaturated soil zone, is a crucial factor in land-atmosphere interactions [1,2]. It plays a key role in determining crop yield and quality [3–6] and is closely linked to many crop stresses such as heat and drought [7]. In open fields, soil moisture fluctuates significantly with changing weather conditions, as rainfall directly influences soil moisture content [8,9]. Accurate measurement and prediction of soil moisture are essential for effective irrigation planning, enabling water application to crops at optimal times and quantities, and improving crop water use efficiency [10].

Methods for measuring soil moisture have advanced significantly over time. The gravimetric method, which directly measures the weight of moisture in soil samples, is accurate but impractical for regional-scale assessments due to its labor-intensive and time-consuming nature [11]. To overcome these limitations, indirect methods using dielectric constant sensors have been developed, which estimate volumetric moisture content by measuring soil permittivity, electrical conductivity, charge storage capacity, or transit time. Time-domain reflectometry is a less invasive but relatively expensive method [12]. Frequency domain reflectometry (FDR), which operates with lower-frequency sensors, is more affordable, simpler, and requires less energy, however it is more susceptible to confounding factors such as temperature, salinity, electrical conductivity, and soil texture [13].

Due to its substantial spatiotemporal variability, soil moisture has been estimated using various methods. One approach uses Richards’ equation, which combines Darcy’s law with the energy balance principle [14]. However, equation-based models can be computationally intensive [15]. Statistical models predict quantities based on patterns learned from historical data. More recently, machine learning and deep learning algorithms have been employed to construct mathematical models that extract information from training datasets and covariates [16]. These approaches are also designed to handle diverse and large datasets, making them potentially valuable tools for operational water management [17].

Long short-term memory (LSTM), a type of machine learning algorithm, has demonstrated excellent performance in time series forecasting, as shown in numerous studies [18–20]. Its architecture was specifically designed to address the long-term gradient vanishing problem in recurrent neural networks (RNN) [21]. The gating mechanism in LSTM enables it to retain or discard information over long sequences, allowing it to learn efficiently from extensive and variable soil moisture datasets and to recognize temporal patterns [22,23]. Furthermore, it can accurately simulate complex nonlinear interactions between variables [24].

Meteorological variables have long been used to predict soil moisture in previous studies [25]. For example, Dolaptsis et al. [26] developed an LSTM model to predict daily reductions in soil moisture content in two maize fields in Turkey, using weather data such as average daily air temperature, total daily solar radiation, average daily relative humidity, and leaf area index. However, models including numerous variables and extensive computations in open-field orchards have a number of practical restrictions that must be considered. Removing some predictors can lead the model to incorrectly weight irrelevant variables, reducing prediction accuracy [27]. Since it is often challenging for growers to consistently collect high-quality data, models should require only a limited number of relevant variables. Furthermore, computational cost must remain low for smart farming technologies to be accessible in both developed and developing countries [28].

In commercial orchards, rainfall provides a significant amount of water supply to the soil and is the primary factor influencing soil moisture dynamics [29]. Hourly rainfall is commonly used as an input variable in modeling [30]. However, individual rainfall event differs in volume, duration, intensity, and the interval between occurrences [31]. Soil moisture responds to the cumulative effect of rainfall, and this response depends on specific characteristics of each event. Therefore, cumulative rainfall may be a more effective input variable in soil moisture prediction models. To better understand how individual rainfall events affect soil moisture, it is essential to segment continuous rainfall records into discrete events.

To the best of our knowledge, there has been minimal study on how to adequately account for rainfall when developing machine learning models that predict soil moisture only on rainfall and soil moisture. We hypothesized that using cumulative rainfall as a meteorological input variable could improve the performance of LSTM models in predicting soil moisture. Therefore, the objectives of this study are as follows: (i) Develop an LSTM model with only rainfall and soil moisture data. (ii) Compare each’s performance with hourly rainfall data and event-based rainfall data as input values. Our findings compared two models designed to forecast soil moisture changes across various time horizons, which enables growers to manage freshwater resources more efficiently and achieve significant economic benefits.

## Materials and methods

### Study area and soil moisture measurements

Soil moisture was determined at a commercial Asian pear orchard located in Naju, Korea (34°58’34.1”N, 126°42’12.7”E).. The slope of surface of study area is 7.5˚ increase. Corresponding author Prof. Sang-Hyun Lee, Director of the Digital Agriculture Research Institute for Horticultural Fruit, was designated as the agricultural manager in the study site, therefore no further permit was required. Data were collected from April to October to focus on the effects of rainfall, excluding potential effects of snow and frost during winter. Topsoil (15–30 cm depth from the soil surface) and subsoil (30–60 cm depth) in the study area are classified as silt loam and silty clay loam, respectively. The soil database collected by the Korean Soil Information System (https://soil.rda.go.kr) was used to compile this soil information, based on granulometric analyses conducted utilizing hydrometer methods.

Soil sampling process for the hydrometer method was conducted as follows. Samples were taken with three replicates in a grid pattern within one-third of the plot area, excluding orchard boundaries and areas compacted by agricultural machinery. Topsoil samples were taken by removing 5 cm from the surface, while subsurface samples were collected 5 cm below the cultivation layer with three replicates collected for each soil layer. Soil samples were air-dried, passed through a 2 mm sieve, oven-dried at 105°C for 18 hours, and weighed to obtain a 50 g sample.

Study area is located in Naju, Korea (34°58’34.1”N, 126°42’12.7”E).

Soil moisture data were recorded separately at depths of 20, 40, and 60 cm from the soil surface at 5-min intervals using frequency domain reflectometry-type soil sensors (RS-ECTH-N01-TR-1, Renke, Sandong, China). Observations were conducted from April to October, 2023 and 2024. During the data collection period, 15,240 soil moisture and 5,136 rainfall data points were collected in 2023, compared with 14,657 and 5,136 data points, respectively, in 2024

Hourly rainfall data for the same periods were obtained from the automatic weather stations operated by the Korea Meteorological Administration (https://www.weather.go.kr). Hourly rainfall data were obtained using a 20-cm diameter tipping-bucket rain gauge, which measures precipitation in 0.5 mm increments.

### Data processing

**Fig 1:**
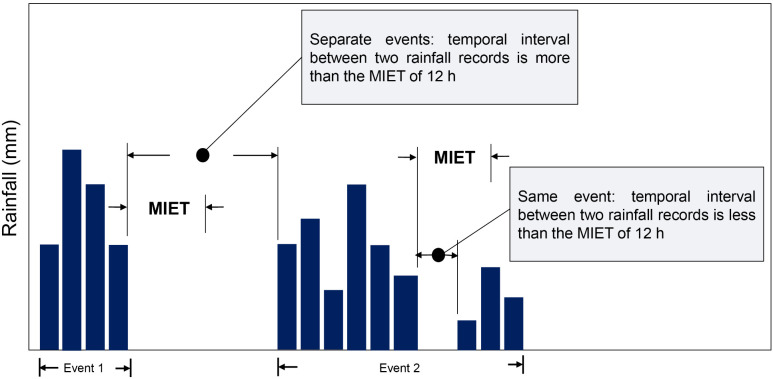
A graphic representation of the minimum inter-event time (MIET).

Cumulative rainfall per event was calculated by applying a minimum inter-event time (MIET) of 12 h to the rainfall dataset. The MIET represents the minimum rain-free period required to distinguish individual rainfall events within a continuous record (Fig 1). According to Lee and Chung [32], a duration of 11–12 h was sufficient to establish rainfall independence. If the gap between two rainfall records was less than 12 h, they were considered part of the same event. If the interval exceeded 12 h, they were treated as separate events.

defined as the minimum rain-free period required to distinguish individual rainfall events within a continuous dataset. If there are 12-hour intervals between rainfall records, they are separated as distinct events; if not, they are grouped together into containers as a single event.

### LSTM model for predicting soil moisture

**Fig 2:**
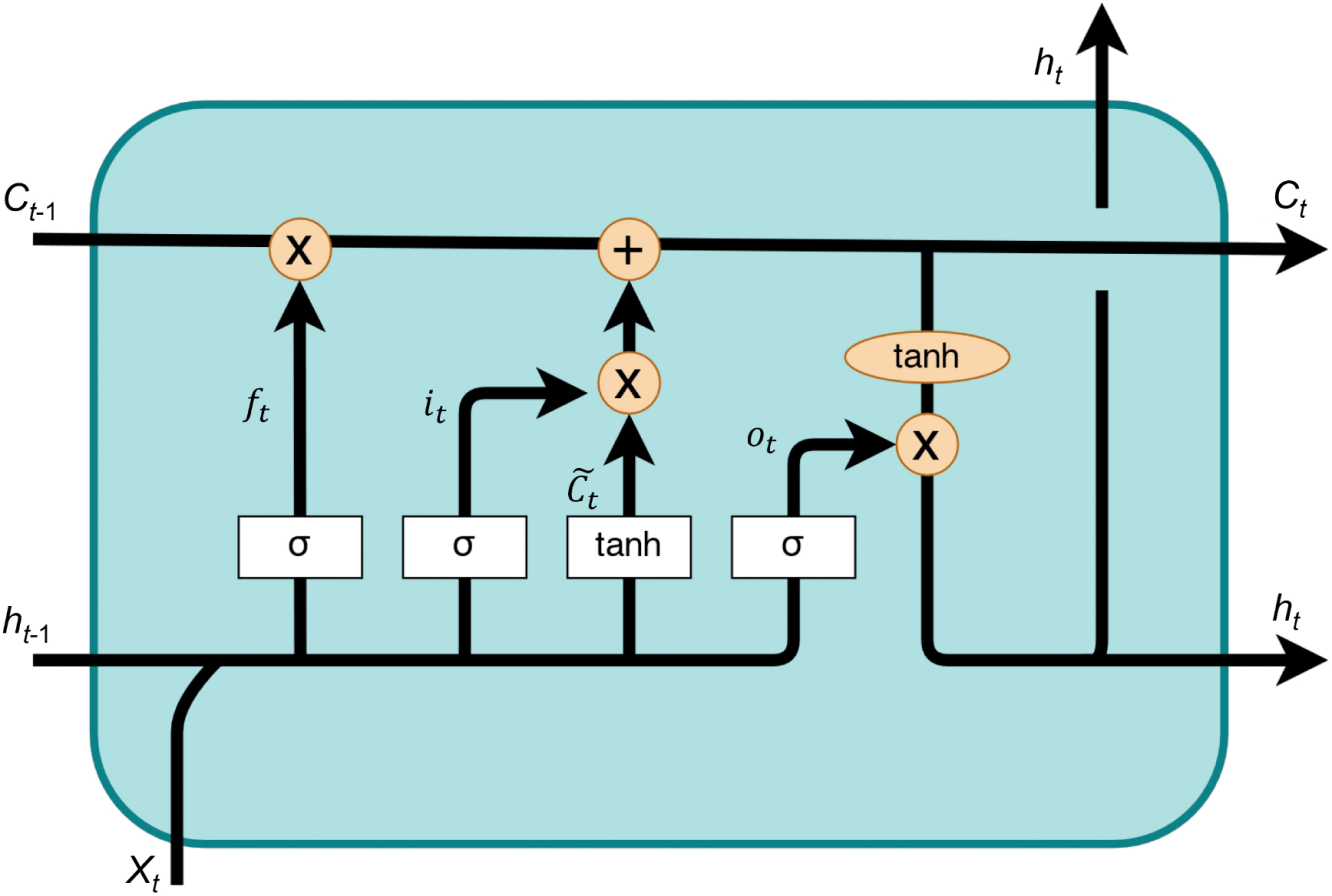
Architecture of LSTM model.


LSTM, a type of RNN, includes memory blocks composed of cells with self-connections that store the temporal state of the network, along with gates (multiplicative units) that regulate the flow of information [33]. Fig 2 and the equations 1–6 show the structure of LSTM, and the equations have a role in each gate [34]. S1 file presents the results of soil moisture prediction using Support Vector Machine and Random Forest, compared to LSTM prediction. This architecture, which determines whether to forget, add, or store information, makes LSTM particularly well-suited for processing time series data [35,36].

$$\begin{array}{c} f_{\text{t}}=\sigma \left(W_{\text{f}}\cdot \left[h_{\text{t}-\text{1}},x_{\text{t}}\right]+b_{\text{f}}\right) \end{array}$$ (1)

$$\begin{array}{c} i_{\text{t}}=\sigma \left(W_{\text{i}}\cdot \left[h_{\text{t}-\text{1}},x_{\text{t}}\right]+b_{\text{i}}\right) \end{array}$$ (2)

$$\begin{array}{c} \tilde{C_{\text{t}}}=\text{tanh}\left(W_{\text{C}}\cdot \left[h_{\text{t}-\text{1}},x_{\text{t}}\right]+b_{\text{C}}\right) \end{array}$$ (3)

$$\begin{array}{c} {\text{C}}_{\text{t}}=f_{\text{t}}\cdot C_{\text{t}-\text{1}}+i_{\text{t}}\cdot \tilde{C_{\text{t}}} \end{array}$$ (4)

$$\begin{array}{c} o_{t}=\sigma \left(W_{o}\cdot \left[h_{\text{t}-\text{1}},x_{\text{t}}\right]+b_{\text{o}}\right) \end{array}$$ (5)

$$\begin{array}{c} h_{\text{t}}=o_{\text{t}}\cdot \text{tanh}\left(C_{\text{t}}\right) \end{array}$$ (6)

Where $f_{\text{t}}$ is the output from the forget gate, $h_{\text{t}-\text{1}}$ is the preceding step, $x_{\text{t}}$ is the current input value, and $b_{\text{f}}$ is a constant bias value. Input gate is consists of two layers. $i_{\text{t}}$ is whether the value needs to be updated or not,$\;\tilde{C_{\text{t}}}$ is a vector of new values that will be added into the LSTM memory. The output gate calculates the final output, where $o_{\text{t}}$ is the output value, and $h_{\text{t}}\;$is its representation as a value between –1 and 1. $W_{\text{f}}$, $W_{\text{i}}$, $W_{\text{C}}$, and $W_{\text{o}}$ are weight matrices.

The preceding step ($h_{\text{t}-\text{1}}$) and the current input value ($x_{\text{t}}$) are consecutively sent through the sigmoid and tanh layers into the forget gate ($f_{\text{t}})$, input gate ($i_{\text{t}}$ and $\tilde{C_{\text{t}}}$), and output gate ($o_{\text{t}}$).

To remove data noise and facilitate effective model learning, all input and output variables were scaled to a range [0, 1] using the MinMaxScaler (https://scikit-learn.org/stable/modules/generated/sklearn.preprocessing.MinMaxScaler.html).

Hyperparameters are selected before LSTM is performed. Grid search optimization is a common method for tuning the parameters of ML classification algorithms, searching exhaustively for the optimal configuration in a predetermined set of hyperparameters as a decision-theoretic method [37,38]. The information on hyperparameters found through grid search is provided in Table 1. Window size, hidden size, num layers, batch size, learning rate were included. The window size is the number of prior time steps supplied into the model at once, indicating how much historical information the LSTM uses to create predictions. Hidden size refers to the dimensionality of the LSTM’s hidden state, which represents the model’s ability to retain and comprehend temporal patterns. Num layers is the number of stacked LSTM layers that determine the depth of sequential feature extraction. Batch size is the number of training samples handled concurrently in a single optimization step. The learning rate is the step size used to update model parameters during training, which determines the speed and stability of convergence. A sensitivity analysis of hyperparameters was conducted. Optimization parameters, such as the learning rate, exhibited relatively clear optimal values regardless of the forecasting time horizon. However, structural parameters, such as the window Size, exhibited contrasting trends depending on the forecasting time point. The preference for small window sizes in event-based rainfall long-term predictions is that, given the physical response time of soil moisture, excessive previous data might behave as noise, raising forecast uncertainty. The number of model input variables was adjusted according to the window size, using two variables: previous soil moisture values and either hourly rainfall or event-based rainfall.

**Table 1:** Hyperparameters for estimating soil moisture.

Soil depth	Time horizon	Hourly rainfall					Event-based rainfall				
		Window size	Hidden size	Num layers	Batch size	Learning rate	Window size	Hidden size	Num layers	Batch size	Learning rate
20 cm	t + 1	24	128	2	64	0.001	24	128	2	64	0.001
	t + 3	24	256	2	128	0.001	24	256	2	64	0.001
	t + 6	24	128	2	64	0.001	24	256	2	64	0.001
	t + 12	24	256	1	64	0.0005	24	256	2	64	0.0005
40 cm	t + 1	24	128	2	128	0.001	24	128	2	64	0.001
	t + 3	24	256	2	128	0.001	24	128	2	64	0.0005
	t + 6	24	128	1	64	0.001	24	256	1	64	0.0005
	t + 12	24	256	2	64	0.0005	24	256	2	64	0.0005
60 cm	t + 1	12	256	2	128	0.001	12	256	2	128	0.001
	t + 3	24	256	2	128	0.001	24	256	1	64	0.001
	t + 6	24	128	1	64	0.0005	24	128	2	64	0.001
	t + 12	24	256	2	64	0.0005	24	256	2	64	0.0005

Hourly rainfall and event-based rainfall are either raw data collected in AWS at 1-hour intervals, or data classified as different rainfall events by MIET, which classifies 12-hour periods of no rainfall between rainfall records as different events after cumulative sum processing. The window size is the number of prior time steps supplied into the model at once, indicating how much historical information the LSTM uses to create predictions. Hidden size refers to the dimensionality of the LSTM’s hidden state, which represents the model’s ability to retain and comprehend temporal patterns. Num layers is the number of stacked LSTM layers that determine the depth of sequential feature extraction. Batch size is the number of training samples handled concurrently in a single optimization step. The learning rate is the step size used to update model parameters during training, which determines the speed and stability of convergence.

The training dataset was split into train 1 and train 2 as follows: train 1 from April 1, 2023, to October 31, 2023, and train 2 from April 1, 2024, to May 10, 2024. Data from May 11, 2024, to June 15, 2024, were used for validation. The test dataset was divided into two parts: test 1 from June 16, 2024 to August 16, 2024, and test 2 from August 23, 2024 to October 31, 2024.

‘t’ stands for the present, and the model inputs include rainfall data and prior soil moisture data, with the goal of predicting soil moisture at four forecast time horizons ‘t + 1’ (i.e., 1 h ahead), ‘t + 3’, ‘t + 6’, ‘t + 12’. Hourly rainfall data and event-based rainfall data were used as input variables for the model, respectively, and the prediction performance of the constructed model was compared.

### Model evaluation

Model performance in predicting soil moisture was evaluated using mean absolute error (MAE), root mean square error (RMSE), and normalized RMSE (nRMSE), calculated as follows:


$$\begin{array}{c} \text{MAE }=\frac{\text{1}}{\text{N}}\sum_{\text{i}=\text{1}}^{\text{N}}\left|{\hat{\text{x}}}_{\text{i}}-{\text{x}}_{\text{i}}\right| \end{array}$$ (7)

$$\begin{array}{c} \text{RMSE}=\sqrt{\frac{\text{1}}{\text{N}}\sum_{\text{i}=\text{1}}^{\text{N}}{\left({\hat{\text{x}}}_{\text{i}}-{\text{x}}_{\text{i}}\right)}^{\text{2}}} \end{array}$$ (8)

$$\begin{array}{c} \text{nRMSE}=\frac{\text{RMSE}}{{\text{x}}_{\text{max}}-{\text{x}}_{\text{min}}} \end{array}$$ (9)

where N is the total number of observations, ${\text{x}}_{\text{i}}$ and $\hat{\text{x}}$ are the observed and predicted values for the 𝕚_th_ observation, respectively, and ${\text{x}}_{\text{max}}$ and ${\text{x}}_{\text{min}}$ are the maximum and minimum observed values, respectively.

The MAE represents the average of the absolute distance between the observed and predicted values, while the RMSE and nRMSE reflect the average magnitude of these difference, giving greater weight to larger errors [39]. Lower MAE, RMSE, and nRMSE values indicate better model performance, signifying a closer fit to the observed data and more accurate predictions.

## Results

### Rainfall and soil moisture

**Fig 3:**
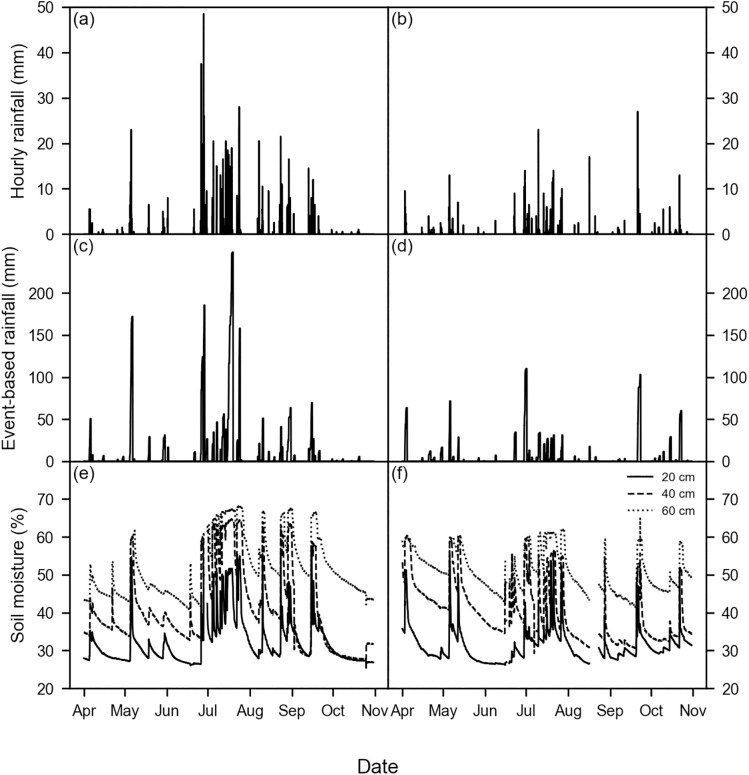
Hourly rainfall, event-based rainfall, and soil moisture in 2023 and 2024.

Hourly rainfall, event-based rainfall, and soil moisture content were monitored in both 2023 and 2024 (Fig 3) in an Asian pear orchard. Total rainfall from April to October was 1,679.5 mm in 2023 (Fig 3A) and 844 mm in 2024 (Fig 3B). The highest hourly rainfall in 2023 was 48.5 mm, recorded on June 28. During the 2024 monitoring period, the highest hourly rainfall of 27 mm recorded on September 21. The number of rainfall events was 47 in 2023 (Fig 3C) and 49 in 2024 (Fig 3D). A notable rainfall event of 248.5 mm was recorded over 96 h beginning on July 14 in 2023 (Fig 3C), with frequent rainfall events from late June through late July (Fig 3C and 3D).

Hourly rainfall data (a, b) were collected at 1-h intervals from an automatic weather station located near the orchard. Event-based rainfall data (c, d) were identified using the minimum inter-event time (MIET) criterion. Hourly soil moisture content at depths of 20, 40, and 60 cm (e, f) below the surface in 2023 (a, c, e) and 2024 (b, d, f) is represented by solid line, dotted lines, respectively. There was more rainfall in 2023 than in 2024. Soil 60 cm depth holds more water than soil 20 cm depth.

Table 2 shows the soil texture analysis. The topsoil (15–32 cm) was silt loam, consisting of 55.3% silt and 26.9% clay. As the depth increased to the subsoil (32–64 cm), the soil texture shifted to silty clay loam, with clay content increasing to 27.8% and sand content decreasing to 13.5%.

**Table 2:** Laboratory data of typifying pedon.

Soil depth (cm)	Sand (%)	Silt (%)	Clay (%)	Soil Texture (USDA)
15-32	17.7	55.3	26.9	silt loam
32-64	13.5	58.7	27.8	silty clay loam

Soil moisture contents increased immediately following the onset of rainfall (Fig 3E and 3F), reflecting the cumulative effects of successive hourly rainfall events. As soil depth increased, soil moisture responded more gradually to rainfall. In the topsoil (20 cm depth), moisture content fluctuated significantly, even in response to minor rainfall events. In contrast, moisture contents in the subsoil (60 cm depth) remained relatively stable for several days after rainfall ended. In addition, average soil moisture content tended to increase with the soil depth yielding 31.6%, 40.4%, and 51.4% at the 20, 40, and 60 cm depths, respectively.

### Soil moisture prediction with LSTM models

**Fig 4:**
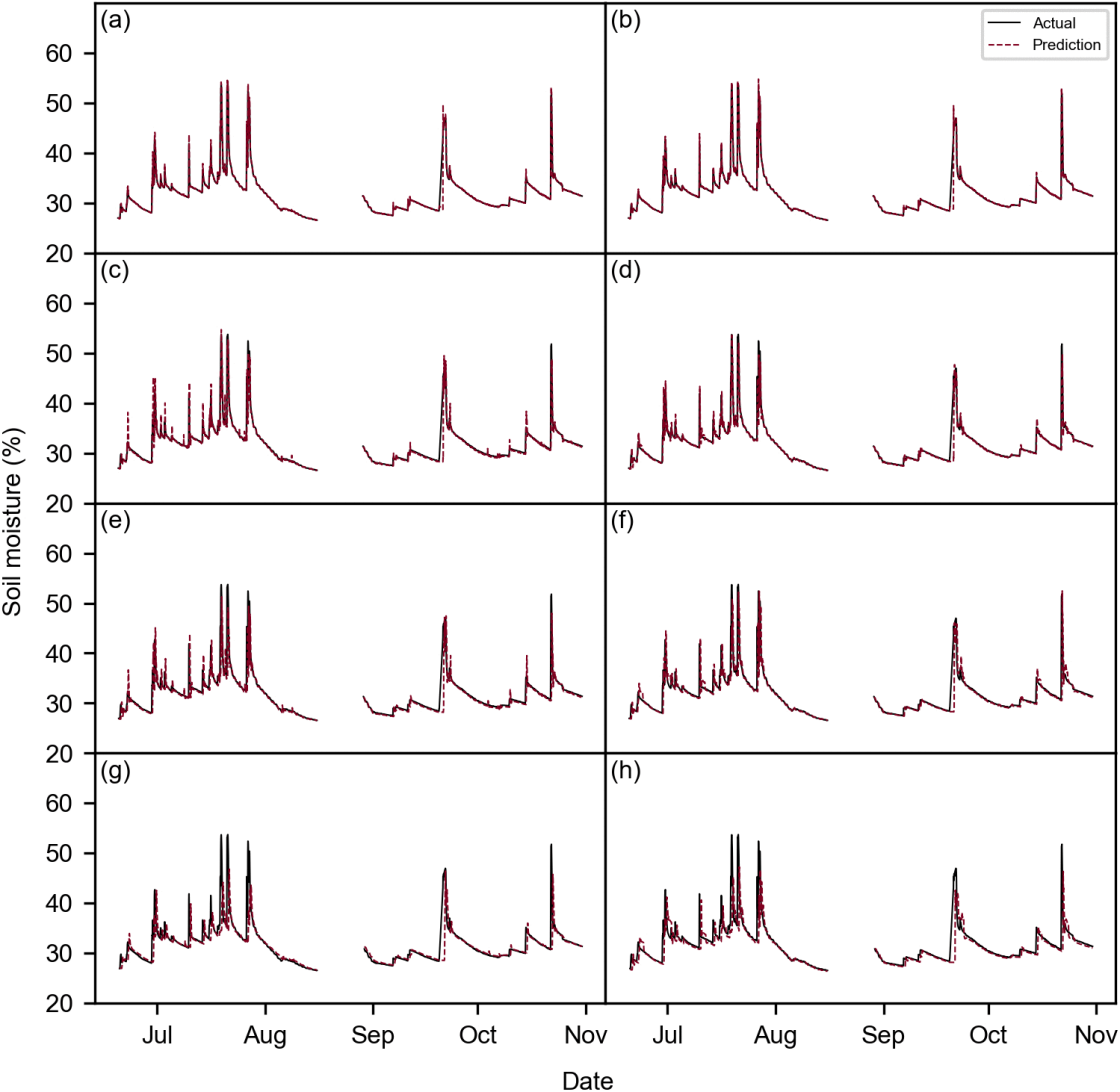
Observed and predicted soil moisture content at a depth of 20 cm. The prediction system was evaluated at four forecast time horizons: ‘t + 1’ (a, b), ‘t + 3’ (c, d), ‘t + 6’ (e, f), and ‘t + 12’ (g, h). Predictions were based on either hourly rainfall data (a, c, e, g) the minimum inter-event time (MIET) or event-based rainfall data with MIET (b, d, f, h). Observed and predicted soil moisture contents are represented by solid and dotted lines, respectively. All comparisons were done over the test period. The model accurately represented temporal patterns at the ‘t + 1’ horizon, however forecasts at longer lead times (‘t + 6’ and ‘t + 12’) consistently underestimated soil moisture fluctuations. Furthermore, short-term spiky variations found in forecasts caused by hourly rainfall inputs (a, c, e, g) were significantly decreased when event-based rainfall data were employed (b, d, f, h).

**Fig 5:**
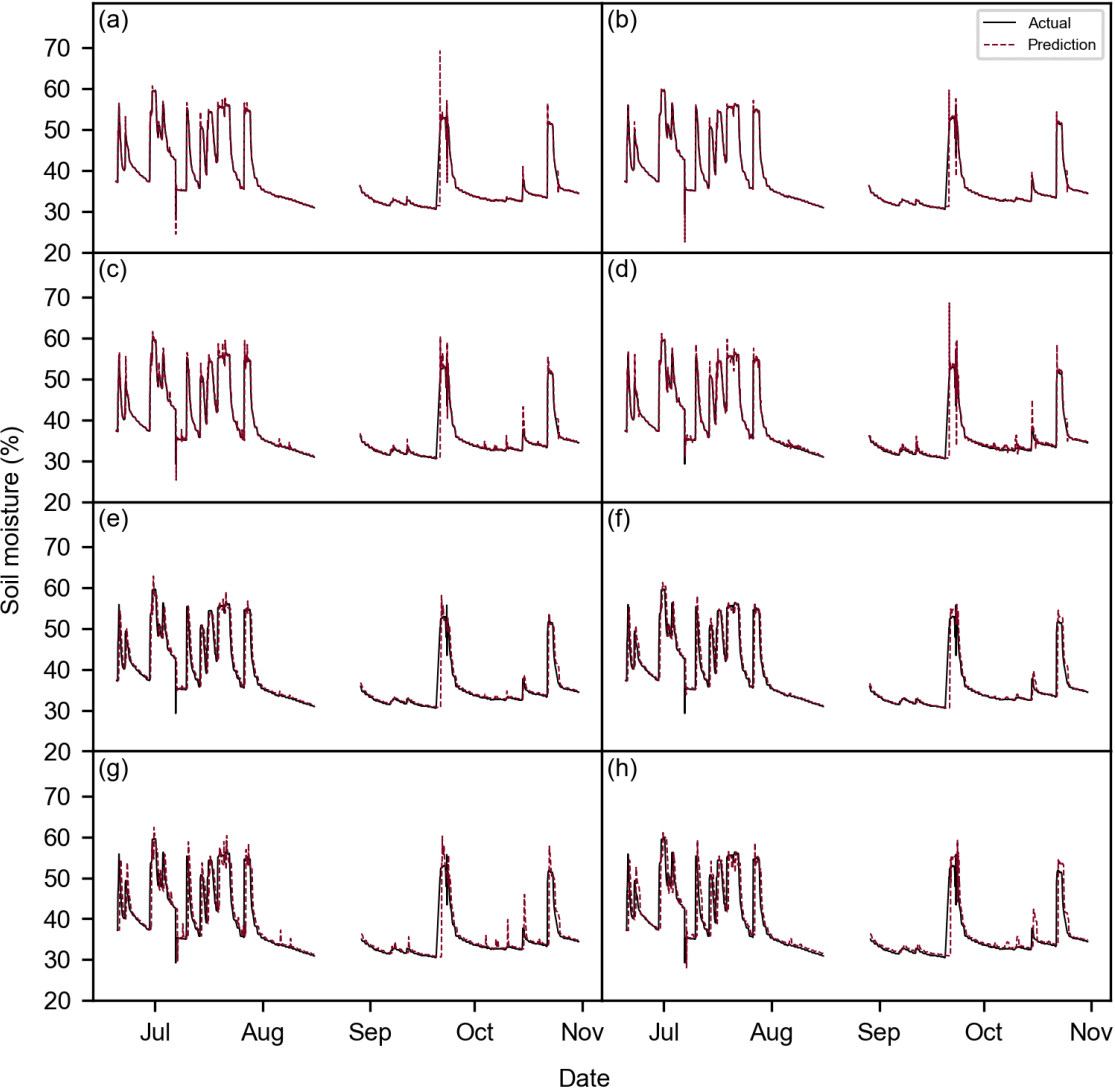
Observed and predicted soil moisture content at a depth of 40 cm. The prediction system was evaluated at four forecast time horizons: ‘t + 1’ (a, b), ‘t + 3’ (c, d), ‘t + 6’ (e, f), and ‘t + 12’ (g, h). Predictions were based on either hourly rainfall data (a, c, e, g) the minimum inter-event time (MIET) or event-based rainfall data with MIET (b, d, f, h). Observed and predicted soil moisture contents are represented by solid and dotted lines, respectively. All comparisons were done over the test period. False sparks occurred most prominently. There was a tendency for overestimation in the event-based rainfall at ‘t + 3’ (c, d).

**Fig 6:**
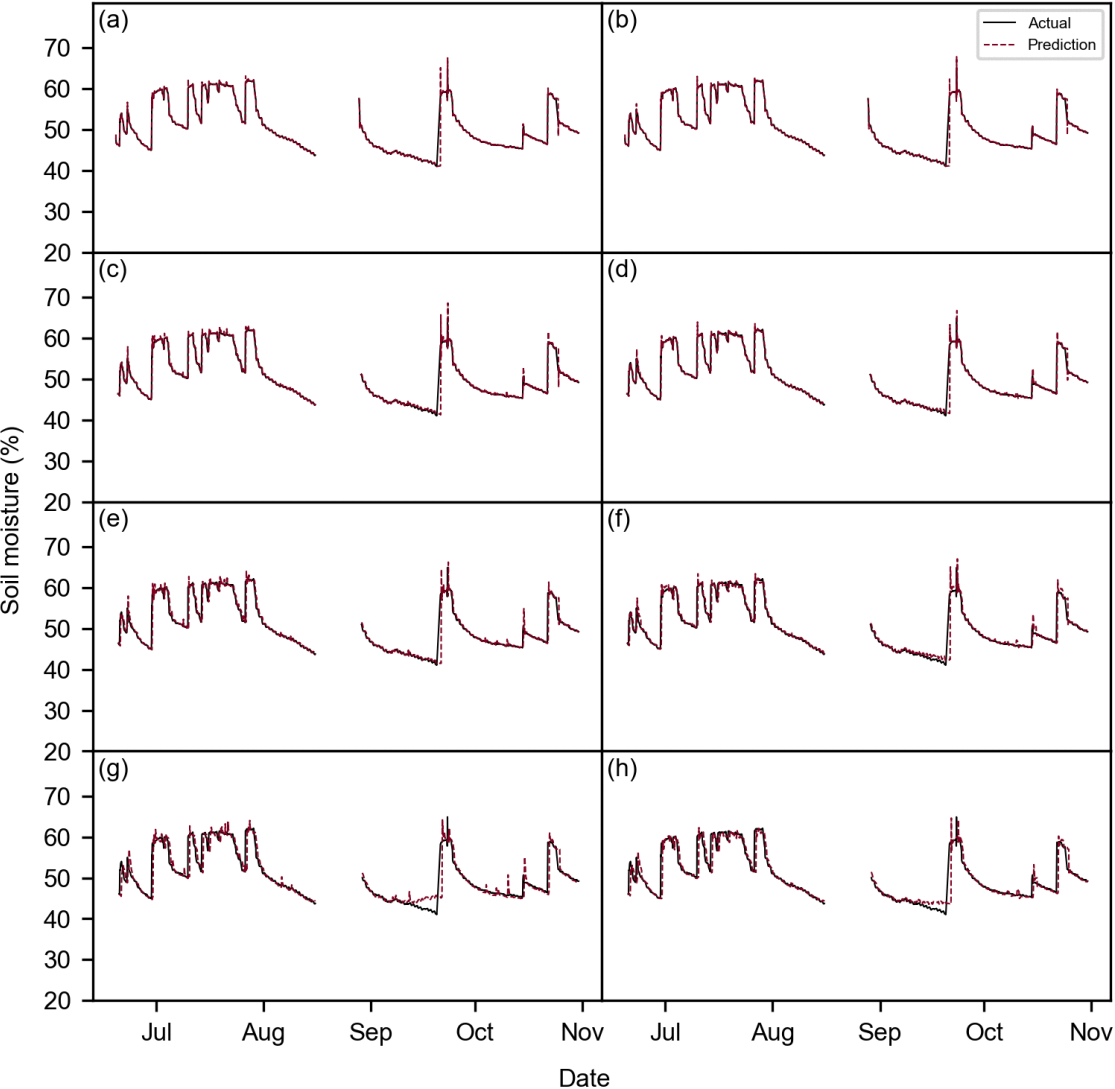
Observed and predicted soil moisture content at a depth of 60 cm. The prediction system was evaluated at four forecast time horizons: ‘t + 1’ (a, b), ‘t + 3’ (c, d), ‘t + 6’ (e, f), and ‘t + 12’ (g, h). Predictions were based on either hourly rainfall data (a, c, e, g) the minimum inter-event time (MIET) or event-based rainfall data with MIET (b, d, f, h). Observed and predicted soil moisture contents are represented by solid and dotted lines, respectively. All comparisons were done over the test period. Although false sparks were small, the model was not suitable for capturing the pattern of soil moisture increasing and then decreasing, notably over the extended prediction time horizon of ‘t + 12’.

LSTM models were developed using rainfall and soil moisture data to predict soil moisture at various soil depths in an Asian pear orchard. The train and validation phases results for 2023 and 2024 in three soil depths are presented in S2 fig, S3 fig, S4 fig, respectively. The performances of the models using hourly and event-based rainfall inputs were compared for predicting soil moisture at depths of 20 (Fig 4), 40 (Fig 5), and 60 cm (Fig 6) from the soil surface. The models were trained to predict soil moisture at various time horizons of ‘t + 1’, ‘t + 3, ‘t + 6’, and ‘t + 12’. The test dataset output varied depending on the type of rainfall input: hourly rainfall data without applying MIET versus event-based rainfall data with MIET, both combined with observed soil moisture data. Short-term predictions (‘t + 1’ and ‘t + 3’) more closely followed the observed soil moisture trends than long-term predictions (‘t + 6’ and ‘t + 12’). The hourly rainfall application model tended to produce false peaks in long-term forecasts, inaccurately predicting increases in soil moisture. These errors were significantly reduced when event-based rainfall data were used.

The model predicting soil moisture at a depth of 40 cm (Fig 5) exhibited the largest prediction error at the time horizon of ‘t + 1’ among all depths (Figs 4–Fig 6). The false peaks observed in the model using hourly rainfall input were reduced in the model using event-based rainfall input. However, unlike at a depth of 20 cm (Fig 4), the model using event-based rainfall input at a depth of 40 cm tended to overestimate peak soil moisture during periods of increase (Fig 5). At a depth of 60 cm (Fig 6), false peaks were less frequent and less pronounced than at a depth of 20 (Fig 4) or 40 cm (Fig 5). However, at a depth of 60 cm, the model using event-based rainfall input was found to be ineffective for capturing the pattern of soil moisture increasing and then decreasing, particularly over the longer prediction time horizon of ‘t + 12’ (Fig 6).

### Performances of LSTM models using hourly and event-based rainfall inputs

Table 3 shows the test period performances of LSTM models using hourly and event-based rainfall inputs for predicting soil moisture were evaluated across various time horizons using MAE, RMSE, and nRMSE values. S5 table shows the LSTM models’ performance results during the training phase.

**Table 3:** Performance comparison of LSTM models using hourly and event-based rainfall inputs in soil moisture prediction at various time horizons at different soil depths in an Asian pear orchard.

Soil depth (cm)	Test Phase	Time	Hourly rainfall			Event-based rainfall		
		horizon	RMSE	MAE	nRMSE	RMSE	MAE	nRMSE
20	Test 1	t + 1	0.75	0.19	0.03	0.74	0.16	0.03
		t + 3	1.81	0.63	0.07	1.93	0.72	0.07
		t + 6	2.62	1.06	0.10	2.41	0.93	0.09
		t + 12	3.02	1.22	0.11	3.10	1.50	0.11
	Test 2	t + 1	0.53	0.10	0.02	0.53	0.08	0.02
		t + 3	1.19	0.32	0.05	1.39	0.45	0.06
		t + 6	1.92	0.53	0.08	1.85	0.47	0.08
		t + 12	2.46	0.69	0.10	2.64	0.95	0.11
40	Test 1	t + 1	0.75	0.21	0.02	0.75	0.21	0.02
		t + 3	1.82	0.72	0.06	1.86	0.71	0.05
		t + 6	3.27	1.57	0.11	3.44	1.74	0.09
		t + 12	4.44	2.47	0.15	4.62	2.58	0.12
	Test 2	t + 1	0.79	0.13	0.03	0.81	0.17	0.03
		t + 3	1.54	0.53	0.06	1.80	0.68	0.07
		t + 6	2.05	0.59	0.08	2.14	0.83	0.08
		t + 12	2.29	1.23	0.09	3.07	1.31	0.12
60	Test 1	t + 1	0.35	0.11	0.02	0.36	0.11	0.02
		t + 3	0.87	0.32	0.05	0.93	0.34	0.05
		t + 6	1.48	0.65	0.08	1.52	0.64	0.08
		t + 12	2.37	1.21	0.13	2.44	1.24	0.13
	Test 2	t + 1	0.60	0.10	0.03	0.59	0.11	0.02
		t + 3	1.02	0.26	0.04	1.01	0.25	0.04
		t + 6	1.49	0.54	0.06	1.46	0.48	0.06
		t + 12	2.07	0.77	0.09	2.09	0.93	0.09

MAE, mean absolute error; RMSE, root mean square error; nRMSE, normalized RMSE. Hourly rainfall and event-based rainfall are either raw data collected in AWS at 1 h intervals, or data classified as different rainfall events by MIET, which classifies 12-hour periods of no rainfall between rainfall records as different events after cumulative sum processing. In time horizon, ‘t’ means present, and model forecasts four future time horizons.

In both models, the soil moisture forecasts at time horizon of ‘t + 1’ yielded the lowest MAE, RMSE, and nRMSE values. When predicting at ‘t + 1’, the highest RMSE values were observed at a depth of 20 cm depth and the lowest RMSE values were observed at a depth of 60 cm depth. Prediction errors increased with longer forecast time horizons, with the highest MAE, RMSE, and nRMSE values occurring at ‘t + 12’. Specifically, the MAE values for soil moisture forecasts at ‘t + 12’ were 1.22, 2.47, and 1.21% at depths of 20, 40, and 60 cm in test 1, respectively, using the hourly rainfall input. When using the event-based rainfall input, the corresponding MAE values were 1.50, 2.58, and 1.24%. Additionally, the RMSE values at ‘t + 12’ were 3.02, 4.44, and 2.37% at depths of 20, 40, and 60 cm, respectively, using the hourly rainfall input. When using the event-based rainfall input, the corresponding RMSE values were 3.10, 4.62, and 2.44%. Scatter plots at three soil depths are presented in S6 fig, S7 fig, and S8 fig.

When hourly rainfall data were used as model input, the prediction residuals at depths of 20 and 40 cm were lower at the shortest time horizon (‘t + 1’) compared to those obtained using the event-based rainfall data. However, at longer time horizons, the prediction residuals increased. At depths of 20 and 60 cm, the prediction residuals remained within an acceptable range relative to the sensor measurement error.

## Discussion

### Rainfall effects on soil moisture

In this study, the pattern of soil moisture increase following rainfall was found to vary with soil depth. This finding indicates that the effect of rainfall on soil moisture is not uniform, but depends on soil depth, consistent with the findings of Park et al. [23], who reported that although rainfall significantly influenced soil moisture, its impact differed by soil depth. This result could be explained from two perspectives. First, the topsoil and subsoil in the examined orchard are classified as silt loam and silty clay loam, respectively. Since clay holds more moisture than silt [40], differences in soil texture might affect water retention capacity. Second, the infiltration depth of rainwater is determined by rainfall intensity [41]. As the rainfall intensity increases, the amplitude of soil moisture variation also increases, in agreement with the findings of Chen et al. [42]. Additionally, in orchards, cultivation practices often loosen the surface soil, promoting more rapid water infiltration in the upper soil layers.

### Performance comparison of LSTM models using hourly and event-based rainfall inputs

In the LSTM model, the RMSE tends to increase with the prediction horizon [43,44] presumably due to the characteristics of the input data. Both input variables were provided at 1-h intervals, thus the ‘t + 1’ prediction, which matches the input time step, showed the best performance. Additionally, while soil moisture responds to accumulated rainfall, hourly rainfall data reflect only the amount occurring within each hour. This can introduce learning errors, as the model may attempt to associate soil moisture changes with incomplete rainfall events. For example, an initial 1 mm of rainfall might have little impact on soil moisture, while an additional 1 mm, once rainfall has accumulated, can significantly increase in soil moisture. As a result, although the residuals between the models using the hourly and event-based rainfall inputs were not significantly different, the model using event-based rainfall input more accurately captured soil moisture peaks, as shown in Figs 4-Fig 6. These improved prediction abilities, particularly in recognizing dynamic soil moisture peaks, provide an algorithmic foundation for the use of smart irrigation systems. Smart irrigation has the ability to increase agricultural efficiency and decrease risks while reducing water waste by up to 95% [45]. The largest prediction residual was observed at a depth of 40 cm, presumably due to the influence of irrigation. As the rainfall data remained at zero, the irregular increases in soil moisture at the onset of irrigation may have contributed to higher prediction errors in the model.

Matei et al. [46] developed a real-time data mining system that collected weather data from ten weather stations to predict next-day soil moisture. The system used the measurements of air temperature, rainfall, and soil temperatures at three soil depths, and soil moisture at a depth of 10 cm. They applied the machine learning algorithms including k-nearest neighbor [47], support vector machine [48], neural networks [49], logistic regression [50], and fast large margin [51], achieving soil moisture prediction accuracies ranging from 64.4 to 74.4%. Similarly, Filipović et al. [52] developed an LSTM-based soil moisture prediction model using daily maximum and minimum temperatures, rainfall, and vapor pressure deficit. Tested on the data from 28 weather stations across Serbia having different soil types at various altitudes, the model demonstrated strong generalization, measured by MAE and mean absolute scaled error. Using the past 60 days of input data, the model predicted soil moisture 3 days ahead, supporting growers in irrigation planning. Although the error measurement values increased when using fewer input days, this result may be specific to the Serbia context.

In this study, the LSTM model was constructed using historical soil moisture and rainfall data estimate soil moisture. The predicted values closely followed the trend of the observed values. These findings contrast with those of Gao et al. [53], who reported that their deep-LSTM soil moisture prediction model, which used multiple meteorological variables including relative humidity, air temperature, rainfall, wind direction, and wind speed, showed the largest RMSE during heavy rainfall periods. A possible reason for this difference is that, unlike the study of Gao et al. [43], the present model used only two variables of rainfall and soil moisture. Since data-driven deep learning models rely greatly on input data [54], careful selection of relevant features can produce models that are faster and easier to train, more robust, and potentially more interpretable [55].

This study demonstrated a strategy for interpreting the soil moisture prediction performance of LSTM models, known for their precision and interpretability. It applied explainable agronomic artificial intelligence by proposing a novel method to enhance the interpretability and reliability of the LSTM models in predicting soil moisture. However, certain limitations remain. This study did not consider the impact of human activities on soil structure, which can be altered by farm machinery-induced soil compaction [56,57] or prolonged wet conditions from irrigation that promote soil hard setting and coalescence [58]. However, the LSTM model developed in this study was not designed to quantify such effects. In addition, although the model demonstrated an acceptable RMSE, future studies should incorporate longer-term data collection to enhance the robustness and reliability of the predictions [59]. Additionally, the performance of the models in soil moisture prediction was evaluated only at a single location. Although the model effectively predicted the topsoil moisture dynamics, future work should extend the predictions across soil profiles and examine spatial variability of soil moisture in the active root zone. Furthermore, microclimate differences, due to the 6.7 km distance between the experimental site and the automatic weather stations for collecting meteorological data, may have affected the prediction accuracy. Future studies should evaluate the generalizability and applicability of the approach using diverse datasets, locations, and input variables. Accurate long-term soil moisture prediction can help develop precision irrigation systems that deliver the right amount of water at the right time. Ultimately, such predictions enable better preparation for potential water shortages.

## Conclusion

Soil moisture is the primary water source for fruit trees in orchards and is replenished mainly by rainfall. To use limited freshwater resources efficiently, it is crucial to analyze and predict how rainfall affects soil moisture. This study developed LSTM models to predict soil moisture in an Asian pear orchard. To improve prediction accuracy, hourly rainfall data were segmented into discrete events using the MIET. During the monitoring period, with the maximum hourly rainfall peaking at 48.5 mm on June 28, 2023, and reaching 27 mm on September 21, 2024. Rainfall-induced changes in moisture were most dynamic in the topsoil, while the subsoil retained added moisture for longer periods. The model showed that soil moisture forecasts at ‘t + 1’ had the lowest MAE and RMSE values across all time horizons. As the forecasting horizon extended to ‘t + 12’, the models still maintained robust performance; specifically, when utilizing hourly rainfall inputs, the MAE values were 1.22%, 2.47%, and 1.21%, while the RMSE values were 3.02%, 4.44%, and 2.37% at depths of 20, 40, and 60 cm, respectively. In comparison, the event-based rainfall inputs yielded slightly higher but acceptable errors at ‘t + 12’, with MAE values of 1.50%, 2.58%, and 1.24%, and RMSE values of 3.10%, 4.62%, and 2.44% across the corresponding depths. Utilizing LSTM models to forecast future soil moisture content in the surface zone, in conjunction with historical event-based rainfall and soil moisture data, can facilitate the accurate detection of changes in soil moisture in open fields more than hourly rainfall. and support the planning of effective irrigation schedules, particularly where freshwater resources are limited. Future research should focus on enhancing soil moisture prediction models by applying them to the entire soil profile.

## Supporting information

S1 File Compared a Support vector machine/Random forest-based soil moisture prediction model with an LSTM prediction model. (DOCX)

S1 Fig Observed and predicted soil moisture contents at a depth of 20 cm in training period. The prediction system was evaluated at four forecast time horizons: ‘t + 1’ (a, b), ‘t + 3’ (c, d), ‘t + 6’ (e, f), and ‘t + 12’ (g, h). Predictions were based on either hourly rainfall data (a, c, e, g) without MIET or event-based rainfall data with the minimum inter-event time (MIET) (b, d, f, h). Observed and predicted soil moisture contents are represented by solid and dotted lines, respectively.
(TIF)

S2 Fig Observed and predicted soil moisture contents at a depth of 40 cm in training period. The prediction system was evaluated at four forecast time horizons: ‘t + 1’ (a, b), ‘t + 3’ (c, d), ‘t + 6’ (e, f), and ‘t + 12’ (g, h). Predictions were based on either hourly rainfall data (a, c, e, g) without MIET or event-based rainfall data with the minimum inter-event time (MIET) (b, d, f, h). Observed and predicted soil moisture contents are represented by solid and dotted lines, respectively.
(TIF)

S3 Fig Observed and predicted soil moisture contents at a depth of 60 cm in training period. The prediction system was evaluated at four forecast time horizons: ‘t + 1’ (a, b), ‘t + 3’ (c, d), ‘t + 6’ (e, f), and ‘t + 12’ (g, h). Predictions were based on either hourly rainfall data (a, c, e, g) without MIET or event-based rainfall data with the minimum inter-event time (MIET) (b, d, f, h). Observed and predicted soil moisture contents are represented by solid and dotted lines, respectively.
(TIF)

S1 Table Performance comparison of LSTM models using hourly and event-based rainfall inputs in soil moisture prediction at various time horizons at different soil depths in train phase. MAE, mean absolute error; RMSE, root mean square error; nRMSE, normalized RMSE. Hourly rainfall and event-based rainfall are either raw data collected in AWS at 1 h intervals, or data classified as different rainfall events by MIET, which classifies 12-hour periods of no rainfall between rainfall records as different events after cumulative sum processing. In time horizon, ‘t’ means present, and model forecasts four future time horizons.
(XLSX)

S4 Fig Scatter plots between observed and predicted soil moisture contents at a depth of 20 cm in test period. ‘t + 1’ (a, b), ‘t + 3’ (c, d), ‘t + 6’ (e, f), and ‘t + 12’ (g, h). Predictions were based on either hourly rainfall data (a, c, e, g) without MIET or event-based rainfall data with the minimum inter-event time (MIET) (b, d, f, h). Dashed diagonal line indicates a 1:1 relationship between measured and predicted soil moisture.
(TIF)

S5 Fig Scatter plots between observed and predicted soil moisture contents at a depth of 40 cm in test period. ‘t + 1’ (a, b), ‘t + 3’ (c, d), ‘t + 6’ (e, f), and ‘t + 12’ (g, h). Predictions were based on either hourly rainfall data (a, c, e, g) without MIET or event-based rainfall data with the minimum inter-event time (MIET) (b, d, f, h). Dashed diagonal line indicates a 1:1 relationship between measured and predicted soil moisture.
(TIF)

S6 Fig Scatter plots between observed and predicted soil moisture contents at a depth of 60 cm in test period. ‘t + 1’ (a, b), ‘t + 3’ (c, d), ‘t + 6’ (e, f), and ‘t + 12’ (g, h). Predictions were based on either hourly rainfall data (a, c, e, g) without MIET or event-based rainfall data with the minimum inter-event time (MIET) (b, d, f, h). Dashed diagonal line indicates a 1:1 relationship between measured and predicted soil moisture.
(TIF)
