# Supplementary material for: Development of long short-term memory models using rainfall and soil moisture to predict soil moisture dynamics
Source: PLoS One. 2026 Jul 21;21(7):e0353268. doi: 10.1371/journal.pone.0353268 (PMC13387561; doi:10.1371/journal.pone.0353268)
Supplement: S1 File — (DOCX) [file pone.0353268.s001.docx]

## SVR and RF model for predicting soil moisture

Information about the hyperparameters used to develop the Support Vector Regression (SVR) model is provided in Table A. C is a regularization constant that determines the trade-off between maximizing margin and minimizing classification error by punishing misclassified or margin-violating data. The C value ranges from 1 to 100, and the Gamma value was trained using scale and 0.1.

**Table A. SVR hyperparameters for estimating soil moisture.**

| Soil depth | Time horizon | Hourly rainfall | | Event-based rainfall | |
| --- | --- | --- | --- | --- | --- |
|  |  | C | Gamma | C | Gamma |
| 20 cm | t+1 | 100 | scale | 100 | scale |
|  | t+3 | 100 | scale | 10 | 0.1 |
|  | t+6 | 10 | 0.1 | 10 | 0.1 |
|  | t+12 | 10 | 0.1 | 1 | scale |
| 40 cm | t+1 | 100 | scale | 1 | 0.1 |
|  | t+3 | 10 | scale | 100 | 0.1 |
|  | t+6 | 10 | scale | 10 | 0.1 |
|  | t+12 | 10 | 0.1 | 10 | 0.1 |
| 60 cm | t+1 | 100 | scale | 100 | 0.1 |
|  | t+3 | 100 | 0.1 | 10 | 0.1 |
|  | t+6 | 10 | 0.1 | 10 | 0.1 |
|  | t+12 | 1 | 0.1 | 1 | 0.1 |

Hourly rainfall and event-based rainfall are either raw data collected in AWS at 1-hour intervals, or data classified as different rainfall events by MIET, which classifies 12-hour periods of no rainfall between rainfall records as different events after cumulative sum processing. C is a regularization constant that determines the trade-off between maximising margin and minimising classification error by punishing misclassified or margin-violating data.

Information about the hyperparameters used to develop the Random Forest (RF) model is provided in Table B. The max_depth is maximum depth of the tree; min_samples_split is minimum number of samples to be split; and n_estimators is the number of trees.

**Table B. RF hyperparameters for estimating soil moisture.**

| Soil depth | Time horizon | Hourly rainfall | | | Event-based rainfall | | | |
| --- | --- | --- | --- | --- | --- | --- | --- | --- |
|  |  | max_depth | min_samples_split | n_estimators | | max_depth | min_samples_split | n_estimators |
| 20 cm | t+1 | 10 | 2 | 100 | | 20 | 2 | 100 |
|  | t+3 | 10 | 2 | 200 | | 10 | 2 | 200 |
|  | t+6 | 10 | 5 | 100 | | 10 | 5 | 100 |
|  | t+12 | 10 | 5 | 200 | | 20 | 5 | 200 |
| 40 cm | t+1 | None | 5 | 100 | | 10 | 5 | 100 |
|  | t+3 | None | 5 | 100 | | 10 | 5 | 100 |
|  | t+6 | None | 5 | 100 | | 20 | 5 | 200 |
|  | t+12 | 10 | 2 | 200 | | None | 5 | 100 |
| 60 cm | t+1 | None | 5 | 200 | | 10 | 5 | 100 |
|  | t+3 | 10 | 5 | 100 | | None | 5 | 200 |
|  | t+6 | 20 | 5 | 100 | | None | 2 | 200 |
|  | t+12 | None | 5 | 100 | | None | 2 | 100 |

Hourly rainfall and event-based rainfall are either raw data collected in AWS at 1-hour intervals, or data classified as different rainfall events by MIET, which classifies 12-hour periods of no rainfall between rainfall records as different events after cumulative sum processing. The max_depth is maximum depth of the tree; min_samples_split is minimum number of samples to be split; and n_estimators is the number of trees.

## SVR and RF model for predicting soil moisture





**Fig A.** **Observed and predicted soil moisture content at a depth of 20 cm.** The prediction system was evaluated at four forecast time horizons: ‘t + 1’ (a, b), ‘t + 3’ (c, d), ‘t + 6’ (e, f), and ‘t + 12’ (g, h). Predictions were based on either hourly rainfall data (a, c, e, g) the minimum inter-event time (MIET) or event-based rainfall data with MIET (b, d, f, h). Observed and predicted soil moisture contents are represented by solid and red (LSTM), blue (RF), Green (SVR) dotted lines, respectively.





**Fig B.** **Observed and predicted soil moisture content at a depth of 40 cm.** The prediction system was evaluated at four forecast time horizons: ‘t + 1’ (a, b), ‘t + 3’ (c, d), ‘t + 6’ (e, f), and ‘t + 12’ (g, h). Predictions were based on either hourly rainfall data (a, c, e, g) the minimum inter-event time (MIET) or event-based rainfall data with MIET (b, d, f, h). Observed and predicted soil moisture contents are represented by solid and red (LSTM), blue (RF), Green (SVR) dotted lines, respectively.





**Fig C.** **Observed and predicted soil moisture content at a depth of 60 cm.** The prediction system was evaluated at four forecast time horizons: ‘t + 1’ (a, b), ‘t + 3’ (c, d), ‘t + 6’ (e, f), and ‘t + 12’ (g, h). Predictions were based on either hourly rainfall data (a, c, e, g) the minimum inter-event time (MIET) or event-based rainfall data with MIET (b, d, f, h). Observed and predicted soil moisture contents are represented by solid and red (LSTM), blue (RF), Green (SVR) dotted lines, respectively.

Fig A, Fig B, and Fig C illustrate the test findings for SVR and RF compared to LSTM over three soil layers, respectively. SVR overestimated soil moisture throughout all time horizons. The frequency of sparking was lower in distant-term projections than in short-term predictions, and this mistake was minimized when event-based rainfall was utilized as an input variable between July and August, when rainfall was abundant.

RF captured the trend, however sparking occurred more frequently than in the other models while calculating soil moisture at 40 cm. At 60 cm, it tended to underestimate long-term horizons. These findings indicate that these models are less successful than LSTM-based techniques for modeling soil moisture.

## Performances of models using hourly and event-based rainfall inputs

**Table C. SVR for estimating soil moisture.**

| Soil depth (cm) | Test | Time | Hourly rainfall | | |  | Event-based rainfall | | |
| --- | --- | --- | --- | --- | --- | --- | --- | --- | --- |
|  | Phase | horizon | RMSE | MAE | nRMSE |  | RMSE | MAE | nRMSE |
| 20 | Test 1 | t + 1 | 2.27 | 1.97 | 8.34 |  | 2.22 | 1.89 | 8.16 |
|  |  | t + 3 | 3.38 | 2.58 | 12.44 |  | 2.99 | 2.75 | 11.01 |
|  |  | t + 6 | 3.22 | 2.84 | 11.84 |  | 3.31 | 2.92 | 12.17 |
|  |  | t + 12 | 3.54 | 2.95 | 13.01 |  | 3.95 | 3.23 | 14.54 |
|  | Test 2 | t + 1 | 2.07 | 1.88 | 8.52 |  | 2.04 | 1.84 | 8.41 |
|  |  | t + 3 | 2.51 | 2.22 | 10.33 |  | 2.78 | 2.62 | 11.46 |
|  |  | t + 6 | 3.00 | 2.72 | 12.34 |  | 3.05 | 2.72 | 12.56 |
|  |  | t + 12 | 3.23 | 2.75 | 13.30 |  | 3.41 | 2.89 | 14.02 |
| 40 | Test 1 | t + 1 | 2.69 | 2.50 | 8.86 |  | 2.80 | 2.67 | 9.22 |
|  |  | t + 3 | 3.14 | 2.68 | 10.32 |  | 3.02 | 2.64 | 9.93 |
|  |  | t + 6 | 3.66 | 2.89 | 12.05 |  | 3.08 | 3.67 | 12.07 |
|  |  | t + 12 | 4.77 | 3.74 | 15.70 |  | 4.82 | 3.78 | 15.86 |
|  | Test 2 | t + 1 | 3.15 | 3.04 | 12.52 |  | 2.73 | 2.66 | 10.83 |
|  |  | t + 3 | 3.22 | 3.06 | 12.79 |  | 3.12 | 2.93 | 12.37 |
|  |  | t + 6 | 3.26 | 2.95 | 12.93 |  | 3.15 | 2.85 | 12.50 |
|  |  | t + 12 | 3.65 | 3.02 | 14.49 |  | 3.65 | 3.01 | 14.50 |
| 60 | Test 1 | t + 1 | 1.39 | 1.18 | 7.51 |  | 1.83 | 1.72 | 9.89 |
|  |  | t + 3 | 1.85 | 1.63 | 10.00 |  | 1.96 | 1.76 | 10.61 |
|  |  | t + 6 | 2.10 | 1.77 | 11.38 |  | 2.18 | 1.87 | 11.78 |
|  |  | t + 12 | 2.59 | 2.05 | 14.02 |  | 2.62 | 2.09 | 14.17 |
|  | Test 2 | t + 1 | 1.36 | 1.09 | 5.69 |  | 1.97 | 1.90 | 8.25 |
|  |  | t + 3 | 1.99 | 1.81 | 8.35 |  | 2.14 | 2.00 | 8.98 |
|  |  | t + 6 | 2.24 | 1.94 | 9.38 |  | 2.28 | 2.00 | 9.55 |
|  |  | t + 12 | 2.59 | 2.09 | 10.87 |  | 2.61 | 2.13 | 10.92 |

MAE, mean absolute error; RMSE, root mean square error; nRMSE, normalized RMSE. Hourly rainfall and event-based rainfall are either raw data collected in AWS at 1 h intervals, or data classified as different rainfall events by MIET, which classifies 12-hour periods of no rainfall between rainfall records as different events after cumulative sum processing. In time horizon, ‘t’ means present, and model forecasts four future time horizons.

**Table D. RF for estimating soil moisture.**

| Soil depth (cm) | Test | | Time | Hourly rainfall | | |  | Event-based rainfall | | |
| --- | --- | --- | --- | --- | --- | --- | --- | --- | --- | --- |
|  | phase | horizon | | RMSE | MAE | nRMSE |  | RMSE | MAE | nRMSE |
| 20 | Test 1 | | t + 1 | 0.92 | 0.25 | 3.38 |  | 0.89 | 0.23 | 3.29 |
|  |  |  | t + 3 | 1.93 | 0.71 | 7.09 |  | 1.86 | 0.67 | 6.85 |
|  |  |  | t + 6 | 2.80 | 1.20 | 10.30 |  | 2.70 | 1.13 | 9.92 |
|  |  |  | t + 12 | 3.30 | 1.63 | 12.15 |  | 3.41 | 1.71 | 12.54 |
|  | Test 2 | | t + 1 | 0.52 | 0.10 | 2.13 |  | 0.51 | 0.10 | 2.12 |
|  |  |  | t + 3 | 1.35 | 0.33 | 5.56 |  | 1.32 | 0.33 | 5.43 |
|  |  |  | t + 6 | 2.11 | 0.66 | 8.68 |  | 2.15 | 0.64 | 8.84 |
|  |  |  | t + 12 | 2.92 | 1.09 | 12.01 |  | 2.78 | 1.04 | 11.45 |
| 40 | Test 1 | | t + 1 | 0.96 | 0.34 | 3.17 |  | 1.53 | 0.70 | 5.04 |
|  |  |  | t + 3 | 2.19 | 1.04 | 7.19 |  | 3.01 | 1.63 | 9.91 |
|  |  |  | t + 6 | 3.39 | 1.77 | 11.16 |  | 3.94 | 2.23 | 12.95 |
|  |  |  | t + 12 | 5.12 | 3.08 | 16.86 |  | 6.62 | 4.02 | 21.78 |
|  | Test 2 | | t + 1 | 0.95 | 0.26 | 3.76 |  | 1.44 | 0.48 | 5.72 |
|  |  |  | t + 3 | 2.07 | 0.85 | 8.20 |  | 2.68 | 1.13 | 10.63 |
|  |  |  | t + 6 | 3.36 | 1.52 | 13.34 |  | 3.92 | 1.75 | 15.55 |
|  |  |  | t + 12 | 4.55 | 2.24 | 18.04 |  | 6.55 | 3.44 | 26.01 |
| 60 | Test 1 | | t + 1 | 0.59 | 0.23 | 3.22 |  | 0.65 | 0.26 | 3.49 |
|  |  |  | t + 3 | 1.11 | 0.51 | 6.01 |  | 1.15 | 0.56 | 6.21 |
|  |  |  | t + 6 | 1.82 | 0.97 | 9.87 |  | 1.85 | 0.97 | 9.99 |
|  |  |  | t + 12 | 2.70 | 1.52 | 14.60 |  | 2.54 | 1.40 | 13.75 |
|  | Test 2 | | t + 1 | 0.62 | 0.13 | 2.60 |  | 0.68 | 0.16 | 2.86 |
|  |  |  | t + 3 | 1.26 | 0.34 | 5.28 |  | 1.27 | 0.36 | 5.32 |
|  |  |  | t + 6 | 1.65 | 0.51 | 6.90 |  | 1.73 | 0.57 | 7.25 |
|  |  |  | t + 12 | 2.27 | 0.79 | 9.52 |  | 0.85 | 2.31 | 9.70 |

MAE, mean absolute error; RMSE, root mean square error; nRMSE, normalized RMSE. Hourly rainfall and event-based rainfall are either raw data collected in AWS at 1 h intervals, or data classified as different rainfall events by MIET, which classifies 12-hour periods of no rainfall between rainfall records as different events after cumulative sum processing. In time horizon, ‘t’ means present, and model forecasts four future time horizons.

Table C and Table D show the test results of SVR and RF. For all models, soil moisture estimates at the 't + 1' time horizon had the lowest MAE, RMSE, and nRMSE values. For predictions at 't + 1', the maximum RMSE value was recorded at a depth of 40 cm, while the lowest was seen at 60 cm. Prediction errors rose with time horizon, peaking at 't + 12' for MAE, RMSE, and nRMSE. When comparing the three models, the SVR model had the highest evaluation index values while the LSTM model had the lowest.
