# Supplementary material for: Development of long short-term memory models using rainfall and soil moisture to predict soil moisture dynamics
Source: PLoS One. 2026 Jul 21;21(7):e0353268. doi: 10.1371/journal.pone.0353268 (PMC13387561; doi:10.1371/journal.pone.0353268)
Supplement: Attachment — Submitted filename: Response to Reviewers.docx [file pone.0353268.s010.docx]

**Response to Editor**

**1. Please ensure that your manuscript meets PLOS ONE's style requirements, including those for file naming.**
• Thank you. We have modified the file names and formats to match PLOS ONE's style requirements.

**2. In your Methods section, please provide additional information regarding the permits you obtained for the work** **Please ensure you have included the full name of the authority that approved the field site access and, if no permits were required, a brief statement explaining why.**
• Thank you for editor’s detailed advice. Corresponding author Prof. Sang-Hyun Lee, Director of the Digital Agriculture Research Institute for Horticultural Fruit, was designated as the agricultural manager in the study site, therefore no further permit was required. We have included this information in lines 111-113.

**3. When completing the data availability statement of the submission form, you indicated that you will make your data available on acceptance. We strongly recommend all authors decide on a data sharing plan before acceptance, as the process can be lengthy and hold up publication timelines. Please note that, though access restrictions are acceptable now, your entire data will need to be made freely accessible if your manuscript is accepted for publication. This policy applies to all data except where public deposition would breach compliance with the protocol approved by your research ethics board. If you are unable to adhere to our open data policy, please kindly revise your statement to explain your reasoning and we will seek the editor's input on an exemption. Please be assured that, once you have provided your new statement, the assessment of your exemption will not hold up the peer review process.**
• Thank you very much for your helpful advice and for taking the time to guide us on this matter. Following your suggestion, all authors carefully discussed the data-sharing plan, all relevant data have been included in this paper and its supplementary information. And the corresponding author has agreed to share the data upon request. We believe that this approach appropriately balances data accessibility and practical considerations, and we hope that it will facilitate data availability without causing any delay in the review process.

**4. We note you have included a table to which you do not refer in the text of your manuscript. Please ensure that you refer to Table 1 in your text; if accepted, production will need this reference to link the reader to the Table.**
• We have carefully revised the manuscript to clarify this point, and Table 1 and Table 2 are now explicitly referenced and explained on lines 168 and line 294, respectively.

**5. If the reviewer's comments include a recommendation to cite specific previously published works, please review and evaluate these publications to determine whether they are relevant and should be cited. There is no requirement to cite these works unless the editor has indicated otherwise.**
• Thank you for your feedback. We have reviewed all reviewer comments and found no recommendations to cite specific works.

**6. The introduction section needs to be extended. Please add a literature review to provide background and context. The research gaps should be clearly identified, and the novelty of the study needs to be explicitly mentioned in the introduction.**

• You have raised an important comment. We agree with you and have incorporated this suggestion throughout the introduction. We added more citations to provide backgrounds. We have clarified the novelty of our study on lines 97-100.

**7. The methodology section requires more detail. Please include additional information about the applied model architecture, explain how the model was tuned, and describe the hyperparameters used. Provide the optimal values of each hyperparameter in a table. Also, include the main equations of the model.**

• Thank you for your kind advice. According to editor's comments, we added the architecture of LSTM model in Fig 3 and added the model’s main equations on lines 148-153. And during the rebuttal, we recognized that the hyperparameters were not optimized during the original model build. Therefore, we've optimized the hyperparameters using grid search and added the results to new Table 1.

**8. Please provide statistical metrics (similar to Table 1) for both the training and testing phases.**

• The editor's comment is correct. We added the model performances to the supplementary Table 1, using the prediction results from the training phases as evaluation metrics, including MAE, RMSE, and nRMSE. Table 2 of the manuscript presents statistical metrics for the testing phases on line 294.

**9. All figures and tables need more explanation and discussion within the text.**
• According to editor’s comment, we added more explanations about all figures and tables captions.

**Response to Reviewers**

**Reviewer 1.**

1. **Figures were not included in the document, which is essential.**

• Thank you for reviewer’s advice. We submitted separate figure files in accordance with PLOS ONE's policy, and they would have been automatically included in the reviewer's PDF.

**2. There is no map of the study area.**
• According to reviewer’s comment, we added map of study area in figure 1.

**3. There are no soil sample samples for the study area, which was limited to one location, according to the researcher.**
• We would like to thank the reviewer for great advice. The study area is a commercial orchard, making it difficult to conduct ongoing soil sampling. This study collated soil information for the region using data from the Soil Environment Information System (SIS) collected by the Republic of Korea's National Institute of Agricultural Sciences (NIAS), a government-run agency with proven reputation. We added more soil analysis information on lines 116-118. And we are aware that the reviewer’s suggestion is essential for experimental designs for specific regions. We have a plan to expand this study to various studies, such as the reviewer’s suggestion.

**4. There is no real comparison between clayey and silty soil texture samples to determine the difference in soil moisture.**
• Thank you for your comments. Soil’s chemical characteristics are immutable and so we refer to literature. Therefore, we revised the sentence and added references to describe the difference between soil types on line 330.

**5. The slope of the surface was not taken into account.**
• According to comment, We added information related to the slope of surface of study site on line 110-111.

**6. It would have been better to compare cultivated and uncultivated soils to obtain better results.**
• Thank you for the reviewer’s suggestion. We agree that comparing cultivated and uncultivated soils would provide more comprehensive results. We plan to address it in future work by expanding the study to diverse soil types and land-use conditions, which we believe will enhance the applicability of our research.

**Reviewer 2**

**1. In the initial part, there is reference in almost every segments ; which is to be optimized**
• Thank you for the reviewer's suggestion. We completely agree with this issue, and we have altered the first section of the Introduction by restructuring and simplifying the references to increase readability and clarity.

**2. Objectives need to be stated precisely (i).. (ii)**
• According to the reviewer’s comment, we separated them into (i), (ii) on lines 101-102.

**3. L129 onwards .. how you arrived at these input set ? need to be elaborated for each case ..sensitivity is analysis is to be added**
• The input set for the LSTM model was determined by tuning window size through grid search. And according to reviewer’s suggestion, We added information about the sensitivity analysis performed on the hyperparameters in lines 175-179.

**4. Soil moisture is already in % .. so what do you mean by normalization in [0,1]?**
• Thank you for the reviewer's advice. Soil moisture is in %, and normalized to [0, 1] means that the data was preprocessed to remove noise and improve predictive power before being trained on the LSTM model. We modified this part in line 165.

**5. What are LSTM control parameters for each case of soil moisture prediction analysis? how you arrived them? sensitivity analysis is missing**
• You’re right. We added information of the parameters on lines 167-183 and table 1.

**6. All plots are black colored ; which is difficult to distinguish ; Also are these for training /testing details are missing to judge or plot are combine d? which is also difficult to figure out performance**
• Thank you for the reviewer's advice. We made all plots black to make the image components accessible to everyone, but we realized that it was difficult to distinguish them. Following the reviewer's advice, we changed the colors to high-contrast colors. We also added a figure for the training and validation period as supporting information fig S1, S2, and S3 .

**7. Also For Table the same comment on partitioning is applicable**
• Based on reviewer comments, we've added table S4, which contains a description of the training set.

**8. Nowadays N number of robust performance evaluation graphical measures are available ; only time series is added .. which is not acceptable**
• According to reviewer’s comment, we added scatter plots in new Supporting information fig S5, S6, and S7 fig.

**9. Above all, only LSTM is used ; how one can assess the creditability of this ? no other ML/DL /other traditional statistical models are used, which is not acceptable**
• Thank you for the reviewer’s thoughtful and constructive comment. We acknowledge the limitations of the study design; however, our primary intention was to effectively utilize rainfall data (e.g., accumulated rainfall events or hourly rainfall) to simulate soil moisture dynamics. We agree with the reviewer’s point regarding the credibility of the LSTM approach, which was not sufficiently emphasized in the previous version of the manuscript. Accordingly, we have discussed other applicable methods for generalizing this study in line 385.

**Reviewer 3**

**1. Line 1-4: Why two titles? Write the appropriate one**
• PLoS One requires both long title and short title. The long title was maintained in the manuscript while the short title was moved to another section of the submission site.

**2. LSTM derived from ?**
• LSTM was developed as a type of RNN as a deep learning algorithm (Hochreiter and Schmidhuber in 1997). We cited the literature on line 75.

**3. Line 25: Specify location. This model only be used in Asian pear orchard ?**
• We expect that the developed LSTM model can be utilized in an Asian pear orchard with a topsoil of silt loam and a subsoil of silt clay loam.

**4. Line 29-30: here you need to explain t + 1 means what ？**
• We added information of ‘t’ and ‘t + 1’ in lines 199-201.

**5. Line 33-34: You must write the duration of days or hours. I think several days is inappropriate**
• Thank you for your comments. We believed that the value varied depending on the total amount, intensity, and duration of each rainfall event. Therefore, the expression was switched on line 34-35 to compare to topsoil.

**6. The introduction is not on specific crop. Kindly revise accordingly to your orchard or which crop. Need to focus on your parameters and also explain horizons 176 of ‘t + 1’, ‘t + 3, ‘t + 6’, and ‘t + 12’etc.**
• You’re right. We agree with your assessment. We added information of crops on lines 45-47 and hyperparameters for LSTM model on lines 166-181. We have modified the information about the time horizon on lines 199-201.

**7. Line 46: You should write regarding fruits production and quality**
• Thank you for your comment. We agreed with you and added fruits information and production in 2025 on lines 45-47 and the effects that water stress can have on crops are described on lines 49-50.

**8. Line 47: Need to focus on your crop or orchards**
• Please see the answers on #7 above.

**9. Line 61-62: Revise it and make one sentence of these two sentences**
• According to the editor’s suggestion, we have modified two sentences into one sentence.

**10. Line 64-65: Provide citations**
• According to the reviewer’s comment, we provided citation on line 66.

**11. Line 69-70: Provide Citation**
• According to the editor’s suggestion, we add the description on lines 73.

**12. Line 76: Provide Citation**
• According to the editor’s suggestion, we add the description on line 79.

**13. Line 85-86: Provide Citation**
• According to the editor’s suggestion, we add the description on line 91.

**14. Line 87-88: Specific characteristics such as ....... and also Provide citation**
• According to the editor’s suggestion, we add the description on line 92-93.

**15. Line 92-97: What is the difference between your work and previous research ?**
• You have raised an important comment. We agree with you and have incorporated this suggestion throughout the introduction. We have clarified the novelty of our study on lines 97-99.

**16. Line 131-132: Kindly explain more**
• Thank you for the reviewer’s great suggestion. We describe the time-series characteristics of the data used to build the model in more detail on lines 199-201, and the description of the input set is shown in Table 1 by window size, time horizon, and input rainfall data type.

**17. I am not able to understand figures because its not clear and presented one year data.**
• Following the reviewer's advice, we changed the colors to high-contrast colors. We describe the splitting of the two years of data we collected into train, validation, and test sets on lines 195-198. We also added a figure for the training and validation period as new Supporting information S1 fig, S2 fig, S3 fig, respectively.

**18. Figures: Poorly presented. No one can understand. Need to improve and it seems to be one year data. Provide two years data. You can also write titles and also treatments to figures.**
• Following the reviewer's advice, we changed the colors to high-contrast colors. We also added a figure for the training and validation period as new Supporting information S1 fig, S2 fig, S3 fig, respectively. According to PlosOne's submission guide, we were unable to include titles or train/test distinctions in the figures, however we included clearer overall figure captions.

**Reviewer 4**

**1. I think the author should put a map of study area in the paper. Also, it would be better if there are a photo of research site.**
• According to a reviewer’s suggestion, we added the photo map of study area in fig 1.

**2. For the FIg 1, why the Y-axis didn't show the axis value ?**
• You have raised an important question. Because Fig. 1 is a simple graphical representation of the Minimum inter-event time (MIET) that distinguishes event-based rainfall, axis values are not shown.

**3. For the FIg 3 to FIg 5, it should to distinguish "Observed" and "Predicted" by a better style, such as use different color.**
• Thank you for reviewer’s kind suggestion. the reviewer's advice, we changed the colors to high-contrast colors.
