# Supplementary material for: Development of long short-term memory models using rainfall and soil moisture to predict soil moisture dynamics
Source: PLoS One. 2026 Jul 21;21(7):e0353268. doi: 10.1371/journal.pone.0353268 (PMC13387561; doi:10.1371/journal.pone.0353268)
Supplement: Attachment — Submitted filename: Response_to_Reviewers_auresp_2.docx [file pone.0353268.s011.docx]

**Response to Reviewers**

**Reviewer 2**

**1.** Nowadays ML/DL applications are abundant.. On advocating the use of LSTM, the performance to be evaluated rigorously with new graphics, comparison of results with multiple tools etc ..the work is so common nowadays and you cant shift for future study .. so comparison of results with those predicted by RF/SVM kind of models (with appropriate control parameter setting), robust graphics (a large number are available) and uncertainty there of is to be assessed .. my comment on such aspects are not addressed

• We appreciate the reviewer's thoughtful and constructive comments. The authors fully agree with the reviewer's point that comparisons with other models are necessary to recommend LSTM. Therefore, in the Supplementary information S1 file in line 147-148, we set the hyperparameters of the RF/SVM model, and we compare the RF/SVM model with the LSTM model. Despite this, compared with others, LSTM is suitable for this long time-series data.

**Reviewer 4**

1. The authors have addressed my comments raised in the previous round of review, and I feel this manuscript is now acceptable for publication in PLOS One.

• We truly appreciate the Reviewer's time, effort, and serious consideration in analyzing our work throughout the review process. We appreciate the Reviewer's constructive comments and valuable recommendations, which helped us improve the manuscript's quality and clarity.
