# Supplementary material for: Development of long short-term memory models using rainfall and soil moisture to predict soil moisture dynamics
Source: PLoS One. 2026 Jul 21;21(7):e0353268. doi: 10.1371/journal.pone.0353268 (PMC13387561; doi:10.1371/journal.pone.0353268)
Supplement: Attachment — Submitted filename: Response_to_Reviewers_auresp_3.docx [file pone.0353268.s012.docx]

**Response to Reviewers**

Reviewer 5

1. [Abstract] It mentions that cumulative rainfall was calculated by applying the minimum inter-event time threshold without indicating what the threshold is. Please indicate it and show how distributive this threshold is over the entire study area.

• We appreciate the reviewer's thoughtful and constructive comments. We would like to clarify that this 12-hour criterion is applied uniformly across the entire study area. In accordance with reviewer's feedback, we have explicitly specified the 12-h threshold for the minimum inter-event time in line 28.

2. [Abstract] Authors mentioned that soil moisture contents increased immediately following the onset of rainfall. Were they expecting something different? How different is this finding from what we know already about rainfall increasing soil moisture content? That should be made clear. Otherwise state that the findings confirm what we know already.

• We appreciate the reviewer's thoughtful and constructive comments. As suggested, we have added a statement clarifying that this insight aligns with previous studies in lines 33–34.

3. [Abstract] Also, the model accuracy, which is base on the RMSE, should be stated including all values of significance for clarity. include also the margins of low/high rainfall amounts obtained in the abstract.

• As following the reviewer’s comment, we added information on rainfall differences on lines 32-33; however, we were unable to specify all results regarding the models’ performance due to the word limit (300 words) of the Abstract section as the guideline of the PLOS ONE journal.

4. [Introduction] I do not see how the opening sentence in the first paragraph is linked to the topic or even the abstract. Please indicate the relation. The set objectives are clear however, the significance and relevance of this study to crop productivity, as explained, is very weak.

• The paragraph has been deleted according to reviewer’s comment.

5. [Materials and Methods] Authors need to be clear on the number of datasets collected for the study from April to October (indicate year). How was the rainfall data obtained and in what measurements? Refer to Line 113 - 114.

• We greatly appreciate your insightful comment. A total of 15,240 and 14,657 soil moisture data points were measured for 2023 and 2024, respectively, along with 5,136 rainfall data points for each year. According to the editor’s suggestion, we add the information of detailed soil moisture and rainfall data observations in lines 129-131 and 133-135.

6. [Materials and Methods] For the collection of soil data (both top and sub), how was the sampling done? Which instruments were used to collect the soil samples, and how many of the samples were collected and in what pattern (grid/diagonal)? How were they stored for the texture analysis? Lines 138 - 140 seems to be isolated. Please link them to the preceding paragraph.

• Thank you for the reviewer’s suggestion. Soil analysis data were collected from the Korean Soil Information System (https://soil.rda.go.kr), which defined the soil sample collecting process as follows: the soil sampling process for the hydrometer method was conducted as follows. Samples were taken with three replicates in a grid pattern within one-third of the plot area, excluding orchard boundaries and areas compacted by agricultural machinery. Topsoil samples were taken by removing 5 cm from the surface, while subsurface samples were collected 5 cm below the cultivation layer with three replicates collected for each soil layer. Soil samples were air-dried, passed through a 2 mm sieve, oven-dried at 105°C for 18 hours, and weighed to obtain a 50 g sample. We added this content in lines 114-122.

7. [Materials and Methods] The narrative for the LSTM model is extensive enough however, it lacks adequate literature to support the narrated activities.

• We agree with reviewer’s comment and have reflected this comment. To support the narrative, we have cited literature on the LSTM architecture in line 158 and lines 178–181.

8. [Results] In addition, reliance on 2 year data alone for the learning and training of the model may cast doubt about the reliability of the study outcome even though the RMSE may project an acceptable outcome.

• Thank you for the reviewer’s thoughtful comment. We agree with you and have added descriptions in lines 403-404.

9. [Results] I do not see any results for the soil texture analysis in relation to the various depths (20cm, 40cm, 60cm). And there was no link of the soil texture to the soil moisture, according to depths for better appreciation of the outcome. Even though there was a discussion on it, there was no results linked to it.

• We agree with the reviewer's comment that linking the soil texture results to the soil moisture outcomes is essential for a better appreciation of the study. To address this, we have explicitly presented the quantitative soil texture data at different depths and average soil moisture contents by incorporating Table 2, in lines 249-251 and 259-260.

10. [Conclusion] The conclusion does not capture the exciting results shown in the manuscript. There was not even a single narration of hourly low/high rainfall amount, what the RMSE/MAE was and what the implications are, for the region. Authors have included general statements on what prediction of soil moisture could detect - Line 400 - 403 - when the research did not look at that. Please conclude on the key findings from this study, highlightinh their implications on soil conservation in the region.

• Thank you for reviewer’s comment. While soil conservation is important, we believe that the most direct and important findings of our soil moisture estimation modeling are those that support the development of smart irrigation by properly utilizing input rainfall data in predicting soil moisture. With this perspective in mind, we have revised the manuscript to emphasize how this model can be utilized as a practical tool for smart irrigation and freshwater conservation. According to the reviewer’s comment, we added the key findings of the study to lines 420-421 and 423-429, and corrected general statements not mentioned in the study in lines 429-432.
