# Supplementary material for: Development of long short-term memory models using rainfall and soil moisture to predict soil moisture dynamics
Source: PLoS One. 2026 Jul 21;21(7):e0353268. doi: 10.1371/journal.pone.0353268 (PMC13387561; doi:10.1371/journal.pone.0353268)
Supplement: Peer review history

# Peer review: Decision Letter 0

**DOI:** 10.1371/journal.pone.0353268.r001

**Authors:** Babak Mohammadi

2 Nov 2025

PONE-D-25-51848

Development of long short-term memory models using rainfall and soil moisture to predict soil moisture dynamics

PLOS ONE

Dear Dr. Chung,

Thank you for submitting your manuscript to PLOS ONE. After careful consideration, we feel that it has merit but does not fully meet PLOS ONE’s publication criteria as it currently stands. Therefore, we invite you to submit a revised version of the manuscript that addresses the points raised during the review process.

Please submit your revised manuscript by Dec 17 2025 11:59PM. If you will need more time than this to complete your revisions, please reply to this message or contact the journal office at plosone@plos.org. When you're ready to submit your revision, log on to https://www.editorialmanager.com/pone/ and select the 'Submissions Needing Revision' folder to locate your manuscript file.

Please include the following items when submitting your revised manuscript:

- A rebuttal letter that responds to each point raised by the academic editor and reviewer(s). You should upload this letter as a separate file labeled 'Response to Reviewers'.
- A marked-up copy of your manuscript that highlights changes made to the original version. You should upload this as a separate file labeled 'Revised Manuscript with Track Changes'.
- An unmarked version of your revised paper without tracked changes. You should upload this as a separate file labeled 'Manuscript'.

If you would like to make changes to your financial disclosure, please include your updated statement in your cover letter. Guidelines for resubmitting your figure files are available below the reviewer comments at the end of this letter.

If applicable, we recommend that you deposit your laboratory protocols in protocols.io to enhance the reproducibility of your results. Protocols.io assigns your protocol its own identifier (DOI) so that it can be cited independently in the future. For instructions see: https://journals.plos.org/plosone/s/submission-guidelines#loc-laboratory-protocols. Additionally, PLOS ONE offers an option for publishing peer-reviewed Lab Protocol articles, which describe protocols hosted on protocols.io. Read more information on sharing protocols at https://plos.org/protocols?utm_medium=editorial-email&utm_source=authorletters&utm_campaign=protocols.

We look forward to receiving your revised manuscript.

Kind regards,

Babak Mohammadi

Academic Editor

PLOS ONE

Journal Requirements:

When submitting your revision, we need you to address these additional requirements.

1. Please ensure that your manuscript meets PLOS ONE's style requirements, including those for file naming. The PLOS ONE style templates can be found at https://journals.plos.org/plosone/s/file?id=wjVg/PLOSOne_formatting_sample_main_body.pdf and https://journals.plos.org/plosone/s/file?id=ba62/PLOSOne_formatting_sample_title_authors_affiliations.pdf

2. In your Methods section, please provide additional information regarding the permits you obtained for the work. Please ensure you have included the full name of the authority that approved the field site access and, if no permits were required, a brief statement explaining why.

3. When completing the data availability statement of the submission form, you indicated that you will make your data available on acceptance. We strongly recommend all authors decide on a data sharing plan before acceptance, as the process can be lengthy and hold up publication timelines. Please note that, though access restrictions are acceptable now, your entire data will need to be made freely accessible if your manuscript is accepted for publication. This policy applies to all data except where public deposition would breach compliance with the protocol approved by your research ethics board. If you are unable to adhere to our open data policy, please kindly revise your statement to explain your reasoning and we will seek the editor's input on an exemption. Please be assured that, once you have provided your new statement, the assessment of your exemption will not hold up the peer review process.

4. We note you have included a table to which you do not refer in the text of your manuscript. Please ensure that you refer to Table 1 in your text; if accepted, production will need this reference to link the reader to the Table.

5. If the reviewer comments include a recommendation to cite specific previously published works, please review and evaluate these publications to determine whether they are relevant and should be cited. There is no requirement to cite these works unless the editor has indicated otherwise.

Additional Editor Comments :

- The introduction section needs to be extended. Please add a literature review to provide background and context. The research gaps should be clearly identified, and the novelty of the study needs to be explicitly mentioned in the introduction.

- The methodology section requires more detail. Please include additional information about the applied model architecture, explain how the model was tuned, and describe the hyperparameters used. Provide the optimal values of each hyperparameter in a table. Also, include the main equations of the model.

- Please provide statistical metrics (similar to Table 1) for both the training and testing phases.

- All figures and tables need more explanation and discussion within the text.

[Note: HTML markup is below. Please do not edit.]

Reviewers' comments:

Reviewer's Responses to Questions

**Comments to the Author**

1. Is the manuscript technically sound, and do the data support the conclusions?

The manuscript must describe a technically sound piece of scientific research with data that supports the conclusions. Experiments must have been conducted rigorously, with appropriate controls, replication, and sample sizes. The conclusions must be drawn appropriately based on the data presented.

Reviewer #1: Yes

Reviewer #2: No

Reviewer #3: Partly

Reviewer #4: Yes

**********

2. Has the statistical analysis been performed appropriately and rigorously?

Reviewer #1: Yes

Reviewer #2: N/A

Reviewer #3: No

Reviewer #4: Yes

**********

3. Have the authors made all data underlying the findings in their manuscript fully available?

The PLOS Data policy requires authors to make all data underlying the findings described in their manuscript fully available without restriction, with rare exception (please refer to the Data Availability Statement in the manuscript PDF file). The data should be provided as part of the manuscript or its supporting information, or deposited to a public repository. For example, in addition to summary statistics, the data points behind means, medians and variance measures should be available. If there are restrictions on publicly sharing data—e.g. participant privacy or use of data from a third party—those must be specified.

Reviewer #1: Yes

Reviewer #2: Yes

Reviewer #3: No

Reviewer #4: Yes

**********

4. Is the manuscript presented in an intelligible fashion and written in standard English?

PLOS ONE does not copyedit accepted manuscripts, so the language in submitted articles must be clear, correct, and unambiguous. Any typographical or grammatical errors should be corrected at revision, so please note any specific errors here.

Reviewer #1: Yes

Reviewer #2: No

Reviewer #3: Yes

Reviewer #4: Yes

**********

5. Review Comments to the Author

Please use the space provided to explain your answers to the questions above. You may also include additional comments for the author, including concerns about dual publication, research ethics, or publication ethics. (Please upload your review as an attachment if it exceeds 20,000 characters)

Reviewer #1: • Figures were not included in the document, which is essential.

• There is no map of the study area.

• There are no soil sample samples for the study area, which was limited to one location, according to the researcher.

• There is no real comparison between clayey and silty soil texture samples to determine the difference in soil moisture.

• The slope of the surface was not taken into account.

• It would have been better to compare cultivated and uncultivated soils to obtain better results.

Reviewer #2: In the initial part, there is reference in almost every segments ; which is to be optimized

Objectives need to be stated precisely (i).. (ii)

L129 onwards .. how you arrived at these input set ? need to be elaborated for each case ..sensitivity is analysis is to be added

Also All such cases like t+1 to t+12, inputs to be summarized in Tabular form

Soil moisture is already in % .. so what do you mean by normalization in [0,1[ ?

What are LSTM control parameters for each case of soil moisture prediction analysis ? how you arrived them ? sensitivity analysis is missing

All plots are black colored ; which is difficult to distinguish ; Also are these for training /testing details are missing to judge or plot are combine d? which is also difficult to figure out performance

Also For Table the same comment on partitioning is applicable

Nowadays N number of robust performance evaluation graphical measures are available ; only time series is added .. which is not acceptable

Above all, only LSTM is used ; how one can assess the creditability of this ? no other ML/DL /other traditional statistical models are used, which is not acceptable

Reviewer #3: The manuscript entitled ‘ Development of long short-term memory models using rainfall and soil moisture topredict soil moisture dynamics’ seems interesting. In current form, I can recommend it in major revision. The author need to revised carefully the following suggestions.

Line 1-4: Why two titles ? Write the appropriate one

LSTM derived from ?

Line 25: Specify location. This model only be used in Asian pear orchard ?

Line 29-30: here you need to explain t + 1 means what �

Line 33-34: You must write the duration of days or hours. I think several days is inappropriate

Introduction

The introduction is not on specific crop. Kindly revise accordingly to your orchard or which crop. Need to focus on your parameters and also explain horizons

176 of ‘t + 1’, ‘t + 3, ‘t + 6’, and ‘t + 12’etc.

Line 46: You should write regarding fruits production and quality

Line 47: Need to focus on your crop or orchards

Line 61-62: Revise it and make one sentence of these two sentences

Line 64-65: Provide citations

Line 69-70: Provide Citation

Line 76: Provide Citation

Line 85-86: Provide Citation

Line 87-88: Specific characteristics such as ....... and also Provide citation

Line 92-97: What is the difference between your work and previous researh ?

Materials and methods

Line 131-132: Kindly explain more

Results : I am not able to understand figures because its not clear and presented one year data.

Figures: Poorly presented. No one can understand. Need to improve and it seems to be one year data. Provide two years data. You can also write titles and also treatments to figures.

Reviewer #4: 1.I think the author should put a map of study area in the paper. Also, it would be better if there are a photo of research site.

2.For the FIg 1, why the Y-axis didn't show the axis value ?

3.For the FIg 3 to FIg 5, it should to distinguish "Observed" and "Predicted" by a better style, such as use different color.

**********

6. PLOS authors have the option to publish the peer review history of their article (what does this mean?). If published, this will include your full peer review and any attached files.

If you choose “no”, your identity will remain anonymous but your review may still be made public.

**Do you want your identity to be public for this peer review?**  For information about this choice, including consent withdrawal, please see our Privacy Policy.

Reviewer #1: **Yes:**  Raheem Hameed Al-Abdan

Reviewer #2: No

Reviewer #3: **Yes:**  Aaqil Khan

Reviewer #4: No

**********

[NOTE: If reviewer comments were submitted as an attachment file, they will be attached to this email and accessible via the submission site. Please log into your account, locate the manuscript record, and check for the action link "View Attachments". If this link does not appear, there are no attachment files.]

To ensure your figures meet our technical requirements, please review our figure guidelines: https://journals.plos.org/plosone/s/figures

You may also use PLOS’s free figure tool, NAAS, to help you prepare publication quality figures: https://journals.plos.org/plosone/s/figures#loc-tools-for-figure-preparation.

NAAS will assess whether your figures meet our technical requirements by comparing each figure against our figure specifications.

# Author comment: Author response to Decision Letter 1

**DOI:** 10.1371/journal.pone.0353268.r002

6 Jan 2026

Reviewer 1.

1. Figures were not included in the document, which is essential.

• Thank you for reviewer’s advice. We submitted separate figure files in accordance with PLOS ONE's policy, and they would have been automatically included in the reviewer's PDF.

2. There is no map of the study area.

• According to reviewer’s comment, we added map of study area in figure 1.

3. There are no soil sample samples for the study area, which was limited to one location, according to the researcher.

• We would like to thank the reviewer for great advice. The study area is a commercial orchard, making it difficult to conduct ongoing soil sampling. This study collated soil information for the region using data from the Soil Environment Information System (SIS) collected by the Republic of Korea's National Institute of Agricultural Sciences (NIAS), a government-run agency with proven reputation. We added more soil analysis information on lines 116-118. And we are aware that the reviewer’s suggestion is essential for experimental designs for specific regions. We have a plan to expand this study to various studies, such as the reviewer’s suggestion.

4. There is no real comparison between clayey and silty soil texture samples to determine the difference in soil moisture.

• Thank you for your comments. Soil’s chemical characteristics are immutable and so we refer to literature. Therefore, we revised the sentence and added references to describe the difference between soil types on line 330.

5. The slope of the surface was not taken into account.

• According to comment, We added information related to the slope of surface of study site on line 110-111.

6. It would have been better to compare cultivated and uncultivated soils to obtain better results.

• Thank you for the reviewer’s suggestion. We agree that comparing cultivated and uncultivated soils would provide more comprehensive results. We plan to address it in future work by expanding the study to diverse soil types and land-use conditions, which we believe will enhance the applicability of our research.

Reviewer 2

1. In the initial part, there is reference in almost every segments ; which is to be optimized

• Thank you for the reviewer's suggestion. We completely agree with this issue, and we have altered the first section of the Introduction by restructuring and simplifying the references to increase readability and clarity.

2. Objectives need to be stated precisely (i).. (ii)

• According to the reviewer’s comment, we separated them into (i), (ii) on lines 101-102.

3. L129 onwards .. how you arrived at these input set ? need to be elaborated for each case ..sensitivity is analysis is to be added

• The input set for the LSTM model was determined by tuning window size through grid search. And according to reviewer’s suggestion, We added information about the sensitivity analysis performed on the hyperparameters in lines 175-179.

4. Soil moisture is already in % .. so what do you mean by normalization in [0,1]?

• Thank you for the reviewer's advice. Soil moisture is in %, and normalized to [0, 1] means that the data was preprocessed to remove noise and improve predictive power before being trained on the LSTM model. We modified this part in line 165.

5. What are LSTM control parameters for each case of soil moisture prediction analysis? how you arrived them? sensitivity analysis is missing

• You’re right. We added information of the parameters on lines 167-183 and table 1.

6. All plots are black colored ; which is difficult to distinguish ; Also are these for training /testing details are missing to judge or plot are combine d? which is also difficult to figure out performance

• Thank you for the reviewer's advice. We made all plots black to make the image components accessible to everyone, but we realized that it was difficult to distinguish them. Following the reviewer's advice, we changed the colors to high-contrast colors. We also added a figure for the training and validation period as supporting information fig S1, S2, and S3 .

7. Also For Table the same comment on partitioning is applicable

• Based on reviewer comments, we've added table S4, which contains a description of the training set.

8. Nowadays N number of robust performance evaluation graphical measures are available ; only time series is added .. which is not acceptable

• According to reviewer’s comment, we added scatter plots in new Supporting information fig S5, S6, and S7 fig.

9. Above all, only LSTM is used ; how one can assess the creditability of this ? no other ML/DL /other traditional statistical models are used, which is not acceptable

• Thank you for the reviewer’s thoughtful and constructive comment. We acknowledge the limitations of the study design; however, our primary intention was to effectively utilize rainfall data (e.g., accumulated rainfall events or hourly rainfall) to simulate soil moisture dynamics. We agree with the reviewer’s point regarding the credibility of the LSTM approach, which was not sufficiently emphasized in the previous version of the manuscript. Accordingly, we have discussed other applicable methods for generalizing this study in line 385.

Reviewer 3

1. Line 1-4: Why two titles? Write the appropriate one

• PLoS One requires both long title and short title. The long title was maintained in the manuscript while the short title was moved to another section of the submission site.

2. LSTM derived from ?

• LSTM was developed as a type of RNN as a deep learning algorithm (Hochreiter and Schmidhuber in 1997). We cited the literature on line 75.

3. Line 25: Specify location. This model only be used in Asian pear orchard ?

• We expect that the developed LSTM model can be utilized in an Asian pear orchard with a topsoil of silt loam and a subsoil of silt clay loam.

4. Line 29-30: here you need to explain t + 1 means what �

• We added information of ‘t’ and ‘t + 1’ in lines 199-201.

5. Line 33-34: You must write the duration of days or hours. I think several days is inappropriate

• Thank you for your comments. We believed that the value varied depending on the total amount, intensity, and duration of each rainfall event. Therefore, the expression was switched on line 34-35 to compare to topsoil.

6. The introduction is not on specific crop. Kindly revise accordingly to your orchard or which crop. Need to focus on your parameters and also explain horizons 176 of ‘t + 1’, ‘t + 3, ‘t + 6’, and ‘t + 12’etc.

• You’re right. We agree with your assessment. We added information of crops on lines 45-47 and hyperparameters for LSTM model on lines 166-181. We have modified the information about the time horizon on lines 199-201.

7. Line 46: You should write regarding fruits production and quality

• Thank you for your comment. We agreed with you and added fruits information and production in 2025 on lines 45-47 and the effects that water stress can have on crops are described on lines 49-50.

8. Line 47: Need to focus on your crop or orchards

• Please see the answers on #7 above.

9. Line 61-62: Revise it and make one sentence of these two sentences

• According to the editor’s suggestion, we have modified two sentences into one sentence.

10. Line 64-65: Provide citations

• According to the reviewer’s comment, we provided citation on line 66.

11. Line 69-70: Provide Citation

• According to the editor’s suggestion, we add the description on lines 73.

12. Line 76: Provide Citation

• According to the editor’s suggestion, we add the description on line 79.

13. Line 85-86: Provide Citation

• According to the editor’s suggestion, we add the description on line 91.

14. Line 87-88: Specific characteristics such as ....... and also Provide citation

• According to the editor’s suggestion, we add the description on line 92-93.

15. Line 92-97: What is the difference between your work and previous research ?

• You have raised an important comment. We agree with you and have incorporated this suggestion throughout the introduction. We have clarified the novelty of our study on lines 97-99.

16. Line 131-132: Kindly explain more

• Thank you for the reviewer’s great suggestion. We describe the time-series characteristics of the data used to build the model in more detail on lines 199-201, and the description of the input set is shown in Table 1 by window size, time horizon, and input rainfall data type.

17. I am not able to understand figures because its not clear and presented one year data.

• Following the reviewer's advice, we changed the colors to high-contrast colors. We describe the splitting of the two years of data we collected into train, validation, and test sets on lines 195-198. We also added a figure for the training and validation period as new Supporting information S1 fig, S2 fig, S3 fig, respectively.

18. Figures: Poorly presented. No one can understand. Need to improve and it seems to be one year data. Provide two years data. You can also write titles and also treatments to figures.

• Following the reviewer's advice, we changed the colors to high-contrast colors. We also added a figure for the training and validation period as new Supporting information S1 fig, S2 fig, S3 fig, respectively. According to PlosOne's submission guide, we were unable to include titles or train/test distinctions in the figures, however we included clearer overall figure captions.

Attachment Submitted filename: Response to Reviewers.docx

# Peer review: Decision Letter 1

**DOI:** 10.1371/journal.pone.0353268.r003

**Authors:** Babak Mohammadi

14 Jan 2026

PONE-D-25-51848R1

Development of long short-term memory models using rainfall and soil moisture to predict soil moisture dynamics

PLOS One

Dear Dr. Chung,

Thank you for submitting your manuscript to PLOS ONE. After careful consideration, we feel that it has merit but does not fully meet PLOS ONE’s publication criteria as it currently stands. Therefore, we invite you to submit a revised version of the manuscript that addresses the points raised during the review process.

Please submit your revised manuscript by Feb 28 2026 11:59PM. If you will need more time than this to complete your revisions, please reply to this message or contact the journal office at plosone@plos.org. When you're ready to submit your revision, log on to https://www.editorialmanager.com/pone/ and select the 'Submissions Needing Revision' folder to locate your manuscript file.

Please include the following items when submitting your revised manuscript:

- A letter that responds to each point raised by the academic editor and reviewer(s). You should upload this letter as a separate file labeled 'Response to Reviewers'.
- A marked-up copy of your manuscript that highlights changes made to the original version. You should upload this as a separate file labeled 'Revised Manuscript with Track Changes'.
- An unmarked version of your revised paper without tracked changes. You should upload this as a separate file labeled 'Manuscript'.

-->If you would like to make changes to your financial disclosure, please include your updated statement in your cover letter. Guidelines for resubmitting your figure files are available below the reviewer comments at the end of this letter.

If applicable, we recommend that you deposit your laboratory protocols in protocols.io to enhance the reproducibility of your results. Protocols.io assigns your protocol its own identifier (DOI) so that it can be cited independently in the future. For instructions see: https://journals.plos.org/plosone/s/submission-guidelines#loc-laboratory-protocols. Additionally, PLOS ONE offers an option for publishing peer-reviewed Lab Protocol articles, which describe protocols hosted on protocols.io. Read more information on sharing protocols at https://plos.org/protocols?utm_medium=editorial-email&utm_source=authorletters&utm_campaign=protocols.

We look forward to receiving your revised manuscript.

Kind regards,

Babak Mohammadi

Academic Editor

PLOS One

Journal Requirements:

If the reviewer comments include a recommendation to cite specific previously published works, please review and evaluate these publications to determine whether they are relevant and should be cited. There is no requirement to cite these works unless the editor has indicated otherwise.

Please review your reference list to ensure that it is complete and correct. If you have cited papers that have been retracted, please include the rationale for doing so in the manuscript text, or remove these references and replace them with relevant current references. Any changes to the reference list should be mentioned in the rebuttal letter that accompanies your revised manuscript. If you need to cite a retracted article, indicate the article’s retracted status in the References list and also include a citation and full reference for the retraction notice.

[Note: HTML markup is below. Please do not edit.]

Reviewers' comments:

Reviewer's Responses to Questions

**Comments to the Author**

1. If the authors have adequately addressed your comments raised in a previous round of review and you feel that this manuscript is now acceptable for publication, you may indicate that here to bypass the “Comments to the Author” section, enter your conflict of interest statement in the “Confidential to Editor” section, and submit your "Accept" recommendation.

Reviewer #2: All comments have been addressed

Reviewer #4: All comments have been addressed

**********

2. Is the manuscript technically sound, and do the data support the conclusions?

The manuscript must describe a technically sound piece of scientific research with data that supports the conclusions. Experiments must have been conducted rigorously, with appropriate controls, replication, and sample sizes. The conclusions must be drawn appropriately based on the data presented.

Reviewer #2: Partly

Reviewer #4: Yes

**********

3. Has the statistical analysis been performed appropriately and rigorously?

Reviewer #2: N/A

Reviewer #4: Yes

**********

4. Have the authors made all data underlying the findings in their manuscript fully available?

The PLOS Data policy requires authors to make all data underlying the findings described in their manuscript fully available without restriction, with rare exception (please refer to the Data Availability Statement in the manuscript PDF file). The data should be provided as part of the manuscript or its supporting information, or deposited to a public repository. For example, in addition to summary statistics, the data points behind means, medians and variance measures should be available. If there are restrictions on publicly sharing data—e.g. participant privacy or use of data from a third party—those must be specified.

Reviewer #2: Yes

Reviewer #4: Yes

**********

5. Is the manuscript presented in an intelligible fashion and written in standard English?

PLOS ONE does not copyedit accepted manuscripts, so the language in submitted articles must be clear, correct, and unambiguous. Any typographical or grammatical errors should be corrected at revision, so please note any specific errors here.

Reviewer #2: Yes

Reviewer #4: Yes

**********

6. Review Comments to the Author

Please use the space provided to explain your answers to the questions above. You may also include additional comments for the author, including concerns about dual publication, research ethics, or publication ethics. (Please upload your review as an attachment if it exceeds 20,000 characters)

Reviewer #2: Nowadays ML/DL applications are abundant.. On advocating the use of LSTM, the performance to be evaluated rigorously with new graphics, comparison of results with multiple tools etc ..the work is so common nowadays and you cant shift for future study .. so comparison of results with those predicted by RF/SVM kind of models (with appropriate control parameter setting), robust graphics (a large number are available) and uncertainty there of is to be assessed .. my comment on such aspects are not addressed

Reviewer #4: The authors have addressed my comments raised in the previous round of review, and I feel this manuscript is now acceptable for publication in PLOS One.

**********

7. PLOS authors have the option to publish the peer review history of their article (what does this mean?). If published, this will include your full peer review and any attached files.

If you choose “no”, your identity will remain anonymous but your review may still be made public.

**Do you want your identity to be public for this peer review?**  For information about this choice, including consent withdrawal, please see our Privacy Policy.

Reviewer #2: No

Reviewer #4: No

**********

[NOTE: If reviewer comments were submitted as an attachment file, they will be attached to this email and accessible via the submission site. Please log into your account, locate the manuscript record, and check for the action link "View Attachments". If this link does not appear, there are no attachment files.]

To ensure your figures meet our technical requirements, please review our figure guidelines: https://journals.plos.org/plosone/s/figures

You may also use PLOS’s free figure tool, NAAS, to help you prepare publication quality figures: https://journals.plos.org/plosone/s/figures#loc-tools-for-figure-preparation.

NAAS will assess whether your figures meet our technical requirements by comparing each figure against our figure specifications.

# Author comment: Author response to Decision Letter 2

**DOI:** 10.1371/journal.pone.0353268.r004

23 Mar 2026

Reviewer 2

1. Nowadays ML/DL applications are abundant.. On advocating the use of LSTM, the performance to be evaluated rigorously with new graphics, comparison of results with multiple tools etc ..the work is so common nowadays and you cant shift for future study .. so comparison of results with those predicted by RF/SVM kind of models (with appropriate control parameter setting), robust graphics (a large number are available) and uncertainty there of is to be assessed .. my comment on such aspects are not addressed

• We appreciate the reviewer's thoughtful and constructive comments. The authors fully agree with the reviewer's point that comparisons with other models are necessary to recommend LSTM. Therefore, in the Supplementary information S1 file in lines 147-148, we set the hyperparameters of the RF/SVM model, and we compare the RF/SVM model with the LSTM model. Despite this, compared with others, LSTM is suitable for this long time-series data.

Reviewer 4

1. The authors have addressed my comments raised in the previous round of review, and I feel this manuscript is now acceptable for publication in PLOS One.

• We truly appreciate the Reviewer's time, effort, and serious consideration in analyzing our work throughout the review process. We appreciate the Reviewer's constructive comments and valuable recommendations, which helped us improve the manuscript's quality and clarity.

Attachment Submitted filename: Response_to_Reviewers_auresp_2.docx

# Peer review: Decision Letter 2

**DOI:** 10.1371/journal.pone.0353268.r005

**Authors:** Babak Mohammadi

27 Apr 2026

PONE-D-25-51848R2

Development of long short-term memory models using rainfall and soil moisture to predict soil moisture dynamics

PLOS One

Dear Dr. Chung,

Thank you for submitting your manuscript to PLOS ONE. After careful consideration, we feel that it has merit but does not fully meet PLOS ONE’s publication criteria as it currently stands. Therefore, we invite you to submit a revised version of the manuscript that addresses the points raised during the review process.

Please submit your revised manuscript by Jun 11 2026 11:59PM. If you will need more time than this to complete your revisions, please reply to this message or contact the journal office at plosone@plos.org. When you're ready to submit your revision, log on to https://www.editorialmanager.com/pone/ and select the 'Submissions Needing Revision' folder to locate your manuscript file.

Please include the following items when submitting your revised manuscript:

- A letter that responds to each point raised by the academic editor and reviewer(s). You should upload this letter as a separate file labeled 'Response to Reviewers'.
- A marked-up copy of your manuscript that highlights changes made to the original version. You should upload this as a separate file labeled 'Revised Manuscript with Track Changes'.
- An unmarked version of your revised paper without tracked changes. You should upload this as a separate file labeled 'Manuscript'.

If you would like to make changes to your financial disclosure, please include your updated statement in your cover letter. Guidelines for resubmitting your figure files are available below the reviewer comments at the end of this letter.

If applicable, we recommend that you deposit your laboratory protocols in protocols.io to enhance the reproducibility of your results. Protocols.io assigns your protocol its own identifier (DOI) so that it can be cited independently in the future. For instructions see: https://journals.plos.org/plosone/s/submission-guidelines#loc-laboratory-protocols. Additionally, PLOS ONE offers an option for publishing peer-reviewed Lab Protocol articles, which describe protocols hosted on protocols.io. Read more information on sharing protocols at https://plos.org/protocols?utm_medium=editorial-email&utm_source=authorletters&utm_campaign=protocols.

As the corresponding author, your ORCID iD is verified in the submission system and will appear in the published article. PLOS supports the use of ORCID, and we encourage all coauthors to register for an ORCID iD and use it as well. Please encourage your coauthors to verify their ORCID iD within the submission system before final acceptance, as unverified ORCID iDs will not appear in the published article. *Only*  the individual author can complete the verification step; PLOS staff *cannot*  verify ORCID iDs on behalf of authors.

We look forward to receiving your revised manuscript.

Kind regards,

Babak Mohammadi

Academic Editor

PLOS One

Journal Requirements:

If the reviewer comments include a recommendation to cite specific previously published works, please review and evaluate these publications to determine whether they are relevant and should be cited. There is no requirement to cite these works unless the editor has indicated otherwise.

Please review your reference list to ensure that it is complete and correct. If you have cited papers that have been retracted, please include the rationale for doing so in the manuscript text, or remove these references and replace them with relevant current references. Any changes to the reference list should be mentioned in the rebuttal letter that accompanies your revised manuscript. If you need to cite a retracted article, indicate the article’s retracted status in the References list and also include a citation and full reference for the retraction notice.

Additional Editor Comments:

I recommend moving Table S4 from the supplementary materials to the main manuscript, as it presents the main findings of the study, including the model’s performance metrics for both the training and testing phases. Additionally, these metrics should be clearly explained and discussed in the main text.

[Note: HTML markup is below. Please do not edit.]

Reviewers' comments:

Reviewer's Responses to Questions

**Comments to the Author**

1. If the authors have adequately addressed your comments raised in a previous round of review and you feel that this manuscript is now acceptable for publication, you may indicate that here to bypass the “Comments to the Author” section, enter your conflict of interest statement in the “Confidential to Editor” section, and submit your "Accept" recommendation.

Reviewer #5: (No Response)

**********

2. Is the manuscript technically sound, and do the data support the conclusions?

The manuscript must describe a technically sound piece of scientific research with data that supports the conclusions. Experiments must have been conducted rigorously, with appropriate controls, replication, and sample sizes. The conclusions must be drawn appropriately based on the data presented.

Reviewer #5: (No Response)

**********

3. Has the statistical analysis been performed appropriately and rigorously?

Reviewer #5: (No Response)

**********

4. Have the authors made all data underlying the findings in their manuscript fully available?

The PLOS Data policy requires authors to make all data underlying the findings described in their manuscript fully available without restriction, with rare exception (please refer to the Data Availability Statement in the manuscript PDF file). The data should be provided as part of the manuscript or its supporting information, or deposited to a public repository. For example, in addition to summary statistics, the data points behind means, medians and variance measures should be available. If there are restrictions on publicly sharing data—e.g. participant privacy or use of data from a third party—those must be specified.

Reviewer #5: Yes

**********

5. Is the manuscript presented in an intelligible fashion and written in standard English?

PLOS ONE does not copyedit accepted manuscripts, so the language in submitted articles must be clear, correct, and unambiguous. Any typographical or grammatical errors should be corrected at revision, so please note any specific errors here.

Reviewer #5: Yes

**********

6. Review Comments to the Author

Please use the space provided to explain your answers to the questions above. You may also include additional comments for the author, including concerns about dual publication, research ethics, or publication ethics. (Please upload your review as an attachment if it exceeds 20,000 characters)

Reviewer #5: Abstract

It mentions that cumulative rainfall was calculated by applying the minimum inter-event time threshold without indicating what the threshold is. Please indicate it and show how distributive this threshold is over the entire study area.

Authors mentioned that soil moisture contents increased immediately following the onset of rainfall. Were they expecting something different? How different is this finding from what we know already about rainfall increasing soil moisture content? That should be made clear. Otherwise state that the findings confirm what we know already.

Also, the model accuracy, which is base on the RMSE, should be stated including all values of significance for clarity. include also the margins of low/high rainfall amounts obtained in the abstract.

Introduction

I do not see how the opening sentence in the first paragraph is linked to the topic or even the abstract. Please indicate the relation. The set objectives are clear however, the significance and relevance of this study to crop productivity, as explained, is very weak.

Materials and Methods

Authors need to be clear on the number of datasets collected for the study from April to October (indicate year). How was the rainfall data obtained and in what measurements? Refer to Line 113 - 114.

For the collection of soil data (both top and sub), how was the sampling done? Which instruments were used to collect the soil samples, and how many of the samples were collected and in what pattern (grid/diagonal)? How were they stored for the texture analysis?

Lines 138 - 140 seems to be isolated. Please link them to the preceding paragraph.

The narrative for the LSTM model is extensive enough however, it lacks adequate literature to support the narrated activities.

In addition, reliance on 2 year data alone for the learning and training of the model may cast doubt about the reliability of the study outcome even though the RMSE may project an acceptable outcome.

Results and Discussion

I do not see any results for the soil texture analysis in relation to the various depths (20cm, 40cm, 60cm). And there was no link of the soil texture to the soil moisture, according to depths for better appreciation of the outcome. Even though there was a discussion on it, there was no results linked to it.

Conclusion

The conclusion does not capture the exciting results shown in the manuscript. There was not even a single narration of hourly low/high rainfall amount, what the RMSE/MAE was and what the implications are, for the region.

Authors have included general statements on what prediction of soil moisture could detect - Line 400 - 403 - when the research did not look at that. Please conclude on the key findings from this study, highlightinh their implications on soil conservation in the region.

**********

7. PLOS authors have the option to publish the peer review history of their article (what does this mean?). If published, this will include your full peer review and any attached files.

If you choose “no”, your identity will remain anonymous but your review may still be made public.

**Do you want your identity to be public for this peer review?**  For information about this choice, including consent withdrawal, please see our Privacy Policy.

Reviewer #5: No

**********

[NOTE: If reviewer comments were submitted as an attachment file, they will be attached to this email and accessible via the submission site. Please log into your account, locate the manuscript record, and check for the action link "View Attachments". If this link does not appear, there are no attachment files.]

To ensure your figures meet our technical requirements, please review our figure guidelines: https://journals.plos.org/plosone/s/figures

You may also use PLOS’s free figure tool, NAAS, to help you prepare publication quality figures: https://journals.plos.org/plosone/s/figures#loc-tools-for-figure-preparation.

NAAS will assess whether your figures meet our technical requirements by comparing each figure against our figure specifications.

# Author comment: Author response to Decision Letter 3

**DOI:** 10.1371/journal.pone.0353268.r006

10 Jun 2026

Reviewer 5

1. [Abstract] It mentions that cumulative rainfall was calculated by applying the minimum inter-event time threshold without indicating what the threshold is. Please indicate it and show how distributive this threshold is over the entire study area.

• We appreciate the reviewer's thoughtful and constructive comments. We would like to clarify that this 12-hour criterion is applied uniformly across the entire study area. In accordance with reviewer's feedback, we have explicitly specified the 12-h threshold for the minimum inter-event time in line 28.

2. [Abstract] Authors mentioned that soil moisture contents increased immediately following the onset of rainfall. Were they expecting something different? How different is this finding from what we know already about rainfall increasing soil moisture content? That should be made clear. Otherwise state that the findings confirm what we know already.

• We appreciate the reviewer's thoughtful and constructive comments. As suggested, we have added a statement clarifying that this insight aligns with previous studies in lines 33–34.

3. [Abstract] Also, the model accuracy, which is base on the RMSE, should be stated including all values of significance for clarity. include also the margins of low/high rainfall amounts obtained in the abstract.

• As following the reviewer’s comment, we added information on rainfall differences on lines 32-33; however, we were unable to specify all results regarding the models’ performance due to the word limit (300 words) of the Abstract section as the guideline of the PLOS ONE journal.

4. [Introduction] I do not see how the opening sentence in the first paragraph is linked to the topic or even the abstract. Please indicate the relation. The set objectives are clear however, the significance and relevance of this study to crop productivity, as explained, is very weak.

• The paragraph has been deleted according to reviewer’s comment.

5. [Materials and Methods] Authors need to be clear on the number of datasets collected for the study from April to October (indicate year). How was the rainfall data obtained and in what measurements? Refer to Line 113 - 114.

• We greatly appreciate your insightful comment. A total of 15,240 and 14,657 soil moisture data points were measured for 2023 and 2024, respectively, along with 5,136 rainfall data points for each year. According to the editor’s suggestion, we add the information of detailed soil moisture and rainfall data observations in lines 129-131 and 133-135.

6. [Materials and Methods] For the collection of soil data (both top and sub), how was the sampling done? Which instruments were used to collect the soil samples, and how many of the samples were collected and in what pattern (grid/diagonal)? How were they stored for the texture analysis? Lines 138 - 140 seems to be isolated. Please link them to the preceding paragraph.

• Thank you for the reviewer’s suggestion. Soil analysis data were collected from the Korean Soil Information System (https://soil.rda.go.kr), which defined the soil sample collecting process as follows: the soil sampling process for the hydrometer method was conducted as follows. Samples were taken with three replicates in a grid pattern within one-third of the plot area, excluding orchard boundaries and areas compacted by agricultural machinery. Topsoil samples were taken by removing 5 cm from the surface, while subsurface samples were collected 5 cm below the cultivation layer with three replicates collected for each soil layer. Soil samples were air-dried, passed through a 2 mm sieve, oven-dried at 105°C for 18 hours, and weighed to obtain a 50 g sample. We added this content in lines 114-122.

7. [Materials and Methods] The narrative for the LSTM model is extensive enough however, it lacks adequate literature to support the narrated activities.

• We agree with reviewer’s comment and have reflected this comment. To support the narrative, we have cited literature on the LSTM architecture in line 158 and lines 178–181.

8. [Results] In addition, reliance on 2 year data alone for the learning and training of the model may cast doubt about the reliability of the study outcome even though the RMSE may project an acceptable outcome.

• Thank you for the reviewer’s thoughtful comment. We agree with you and have added descriptions in lines 403-404.

9. [Results] I do not see any results for the soil texture analysis in relation to the various depths (20cm, 40cm, 60cm). And there was no link of the soil texture to the soil moisture, according to depths for better appreciation of the outcome. Even though there was a discussion on it, there was no results linked to it.

• We agree with the reviewer's comment that linking the soil texture results to the soil moisture outcomes is essential for a better appreciation of the study. To address this, we have explicitly presented the quantitative soil texture data at different depths and average soil moisture contents by incorporating Table 2, in lines 249-251 and 259-260.

10. [Conclusion] The conclusion does not capture the exciting results shown in the manuscript. There was not even a single narration of hourly low/high rainfall amount, what the RMSE/MAE was and what the implications are, for the region. Authors have included general statements on what prediction of soil moisture could detect - Line 400 - 403 - when the research did not look at that. Please conclude on the key findings from this study, highlightinh their implications on soil conservation in the region.

• Thank you for reviewer’s comment. While soil conservation is important, we believe that the most direct and important findings of our soil moisture estimation modeling are those that support the development of smart irrigation by properly utilizing input rainfall data in predicting soil moisture. With this perspective in mind, we have revised the manuscript to emphasize how this model can be utilized as a practical tool for smart irrigation and freshwater conservation. According to the reviewer’s comment, we added the key findings of the study to lines 420-421 and 423-429, and corrected general statements not mentioned in the study in lines 429-432.

Attachment Submitted filename: Response_to_Reviewers_auresp_3.docx

# Peer review: Decision Letter 3

**DOI:** 10.1371/journal.pone.0353268.r007

**Authors:** Babak Mohammadi

22 Jun 2026

Development of long short-term memory models using rainfall and soil moisture to predict soil moisture dynamics

PONE-D-25-51848R3

Dear Dr. Chung,

We’re pleased to inform you that your manuscript has been judged scientifically suitable for publication and will be formally accepted for publication once it meets all outstanding technical requirements.

Within one week, you’ll receive an e-mail detailing the required amendments. When these have been addressed, you’ll receive a formal acceptance letter and your manuscript will be scheduled for publication.

An invoice will be generated when your article is formally accepted. Please note, if your institution has a publishing partnership with PLOS and your article meets the relevant criteria, all or part of your publication costs will be covered. Please make sure your user information is up-to-date by logging into Editorial Manager at Editorial Manager® and clicking the ‘Update My Information' link at the top of the page. For questions related to billing, please contact billing support.

If your institution or institutions have a press office, please notify them about your upcoming paper to help maximize its impact. If they’ll be preparing press materials, please inform our press team as soon as possible -- no later than 48 hours after receiving the formal acceptance. Your manuscript will remain under strict press embargo until 2 pm Eastern Time on the date of publication. For more information, please contact onepress@plos.org.

Kind regards,

Babak Mohammadi

Academic Editor

PLOS One

Additional Editor Comments (optional):

This manuscript is acceptable.

Reviewers' comments:

Reviewer's Responses to Questions

**Comments to the Author**

1. If the authors have adequately addressed your comments raised in a previous round of review and you feel that this manuscript is now acceptable for publication, you may indicate that here to bypass the “Comments to the Author” section, enter your conflict of interest statement in the “Confidential to Editor” section, and submit your "Accept" recommendation.

Reviewer #5: (No Response)

**********

2. Is the manuscript technically sound, and do the data support the conclusions?

The manuscript must describe a technically sound piece of scientific research with data that supports the conclusions. Experiments must have been conducted rigorously, with appropriate controls, replication, and sample sizes. The conclusions must be drawn appropriately based on the data presented.

Reviewer #5: Yes

**********

3. Has the statistical analysis been performed appropriately and rigorously?

Reviewer #5: Yes

**********

4. Have the authors made all data underlying the findings in their manuscript fully available?

The PLOS Data policy requires authors to make all data underlying the findings described in their manuscript fully available without restriction, with rare exception (please refer to the Data Availability Statement in the manuscript PDF file). The data should be provided as part of the manuscript or its supporting information, or deposited to a public repository. For example, in addition to summary statistics, the data points behind means, medians and variance measures should be available. If there are restrictions on publicly sharing data—e.g. participant privacy or use of data from a third party—those must be specified.

Reviewer #5: Yes

**********

5. Is the manuscript presented in an intelligible fashion and written in standard English?

PLOS ONE does not copyedit accepted manuscripts, so the language in submitted articles must be clear, correct, and unambiguous. Any typographical or grammatical errors should be corrected at revision, so please note any specific errors here.

Reviewer #5: Yes

**********

6. Review Comments to the Author

Please use the space provided to explain your answers to the questions above. You may also include additional comments for the author, including concerns about dual publication, research ethics, or publication ethics. (Please upload your review as an attachment if it exceeds 20,000 characters)

Reviewer #5: Abstract

Authors have justified that the omission of accuracy results from the abstract was due to word limitation. However, authors could adjust the texts by working on the long sentences to make room for the accuracy values. The key accuracy values need to be highlighted in the abstract for better model appreciation at the first read.

The others sections have been properly worked on and they make a sound scientific reading.

**********

7. PLOS authors have the option to publish the peer review history of their article (what does this mean?). If published, this will include your full peer review and any attached files.

If you choose “no”, your identity will remain anonymous but your review may still be made public.

**Do you want your identity to be public for this peer review?**  For information about this choice, including consent withdrawal, please see our Privacy Policy.

Reviewer #5: No

**********

# Editor report: Acceptance letter

**DOI:** 10.1371/journal.pone.0353268.r008

**Authors:** Babak Mohammadi

PONE-D-25-51848R3

PLOS One

Dear Dr. Chung,

I'm pleased to inform you that your manuscript has been deemed suitable for publication in PLOS One. Congratulations! Your manuscript is now being handed over to our production team.

At this stage, our production department will prepare your paper for publication. This includes ensuring the following:

* All references, tables, and figures are properly cited

* All relevant supporting information is included in the manuscript submission,

* There are no issues that prevent the paper from being properly typeset

You will receive further instructions from the production team, including instructions on how to review your proof when it is ready. Please keep in mind that we are working through a large volume of accepted articles, so please give us a few days to review your paper and let you know the next and final steps.

Lastly, if your institution or institutions have a press office, please let them know about your upcoming paper now to help maximize its impact. If they'll be preparing press materials, please inform our press team within the next 48 hours. Your manuscript will remain under strict press embargo until 2 pm Eastern Time on the date of publication. For more information, please contact onepress@plos.org.

You will receive an invoice from PLOS for your publication fee after your manuscript has reached the completed accept phase. If you receive an email requesting payment before acceptance or for any other service, this may be a phishing scheme. Learn how to identify phishing emails and protect your accounts at https://explore.plos.org/phishing.

If we can help with anything else, please email us at customercare@plos.org.

Thank you for submitting your work to PLOS ONE and supporting open access.

Kind regards,

PLOS ONE Editorial Office Staff

on behalf of

Dr. Babak Mohammadi

Academic Editor

PLOS One
